# A Causal and interpretable machine learning framework for postcranioplasty risk prediction and surgical decision support

**DOI:** 10.1038/s41746-026-02370-6

**Published:** 2026-01-21

**Authors:** Wenbo Li, Bao Wang, Tianzun Li, Yiwen Ma, Haoyong Jin, Jiangli Zhao, Zhiwei Xue, Nan Su, Yanya He, Jiaqi Shi, Xuchen Liu, Xiaoyang Liu, Tianzi Wang, Jiwei Wang, Chao Li, Can Yan, Yang Ma, Qichao Qi, Xinyu Wang, Weiguo Li, Bin Huang, Donghai Wang, Xuelian Wang, Yan Qu, Xingang Li, Chen Qiu, Ning Yang

**Affiliations:** 1 Department of Neurosurgery, Qilu Hospital, Cheeloo College of Medicine and Institute of Brain and Brain-Inspired Science, Shandong University, Jinan, China; 2 School of Medicine, Cheeloo College of Medicine, Shandong University, Jinan, China; 3 Shandong Key Laboratory of Brain Health and Function Remodeling, Jinan, China; 4 Department of Neurosurgery, Tangdu Hospital, Fourth Military Medical University, Xi’an, China; 5 Center for Frontier Medicine Innovation, Tangdu Hospital, Fourth Military Medical University, Xi’an, China; 6 Department of Neurosurgery, Daping Hospital, Army Military Medical University, Chongqing, China; 7 School of Computer Science, Hangzhou Dianzi University, Hangzhou, China; 8 Department of Critical Care Medicine, Qilu Hospital of Shandong University, Jinan, China; 9 Department of Radiation Oncology, Qilu Hospital of Shandong University, Jinan, China

**Keywords:** Computational biology and bioinformatics, Medical research, Neurology, Risk factors

## Abstract

Cranioplasty is associated with a substantial burden of postoperative complications. In this multicenter study, we developed a machine learning–based clinical decision-support tool to predict the risk of postoperative complications following cranioplasty. A set of nine features was selected for model development. Among the 15 algorithms evaluated, the random forest model demonstrated the best overall performance and was validated on data from both spatial and temporal external cohorts (AUROC = 0.949, internal cross-validation; 0.930, geographical validation; and 0.932, temporal validation). Subgroup analyses by age and sex demonstrated consistently high discriminative performance (lowest AUROC = 0.927) and good calibration (O/E ratio = 1.16, 95% CI: 0.97–1.40). Analysis of causal effects of modifiable intraoperative variables on postoperative complications, with diverse counterfactual explanations and causal inference methods, including double machine learning and the T-learner framework, revealed a protective effect of subcutaneous negative-pressure drainage (ATE = −0.241) and titanium mesh (ATE = −0.191). Finally, we present the model as an accessible web-based tool for individualized, real-time clinical decision-making (http://www.cranioplastycomplicationprediction.top). These findings provide a practical framework for postoperative risk stratification and support the optimization of intraoperative decision-making in cranioplasty.

## Introduction

Decompressive craniectomy is a well-established neurosurgical procedure used to alleviate elevated intracranial pressure caused by conditions such as traumatic brain injury, cerebral infarction, and intracranial hemorrhage (ICH)^1–3^. Although decompressive craniectomy is lifesaving, it results in a cranial defect that leaves the brain vulnerable to mechanical injuries and physiological disturbances^4,5^. To address these risks, cranioplasty is routinely performed to restore cranial integrity. Especially for patients who have undergone hemicraniectomy, cranioplasty is ultimately required, as failure to restore cranial integrity can lead to sinking skin flap syndrome or the syndrome of the trephined. In addition to restoring structural protection, cranioplasty has been shown to improve neurological function and enhance esthetic outcomes, thereby facilitating recovery and promoting psychological well-being^6^.

Although cranioplasty is considered a technically straightforward procedure, it is associated with a relatively high incidence of postoperative complications^7^. Complications include infection, ICH, hydrocephalus, seizures, fluid collection, and pneumocephalus. These adverse events not only prolong hospital stays and increase healthcare costs but also impair recovery and quality of life^8^.

Given these risks, early identification of high-risk patients is crucial for facilitating more efficient use of clinical resources, guiding perioperative management, and ultimately improving outcomes. Currently, there are numerous studies that have investigated the factors associated with postoperative complications following cranioplasty^9–11^. However, these studies have primarily focused on identifying risk factors rather than developing predictive models to forecast complications. As a result, reliable tools for predicting postoperative complications are still lacking, leaving clinicians to rely on empirical judgment and reactive strategies. This limitation substantially reduces the clinical applicability of prior findings. It also underscores the need for robust and interpretable predictive models to support individualized patient care.

In this study, we aimed to develop and validate an explainable machine learning (ML)–based clinical tool for predicting postoperative complications following cranioplasty. By integrating causal and interpretable machine learning into clinical workflows, this work seeks to bridge the gap between predictive modeling and actionable decision support in cranioplasty, ultimately advancing personalized neurosurgical care and improving postoperative outcomes.

## Results

### Population characteristics

**Fig. 1:**
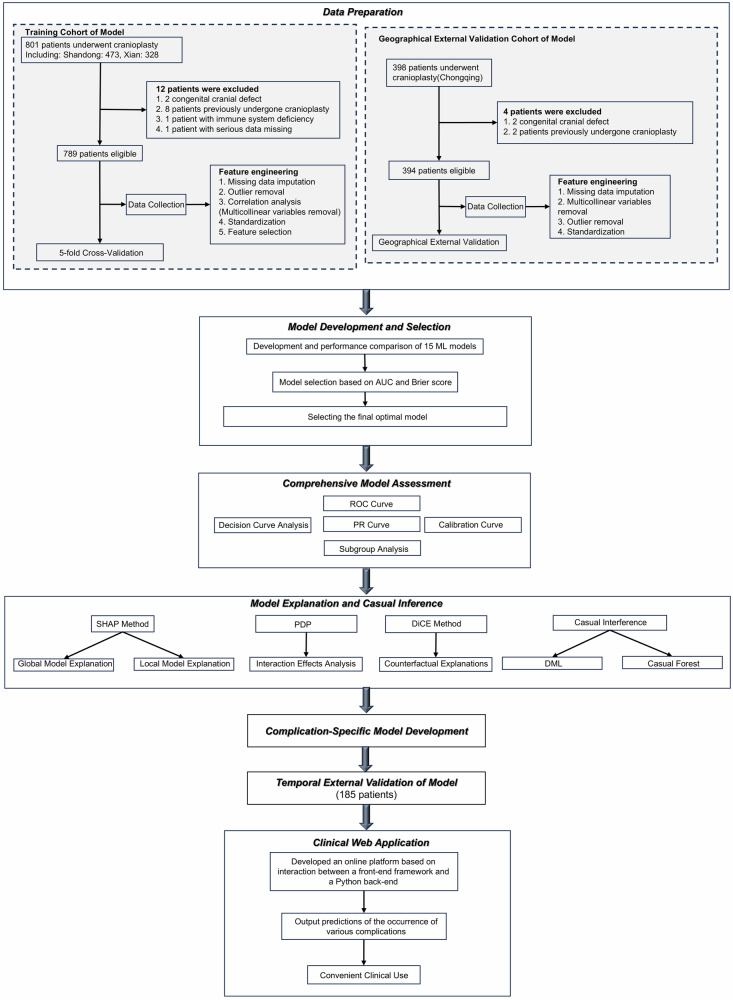
Flow chart of the study design. ML machine learning, ROC curve receiver operating characteristic curve, SHAP SHapley Additive exPlanations, PDP partial dependence plot, DiCE diverse counterfactual explanations, PR curve precision-recall curve, DML double machine learning.

The study design is illustrated in Fig. 1, and baseline characteristics of the derivation cohort (*n* = 789), geographical external validation cohort (*n* = 394), and temporal external validation cohort (*n* = 185) are summarized in Table 1. Postoperative complications occurred in 205 (26.0%) patients in the derivation cohort, 115 (29.2%) in the geographical external validation cohort, and 51 (27.6%) in the temporal external validation cohort. Reoperations due to severe complications were required in 13 patients (1.6%) in the derivation cohort, 8 patients (2.0%) in the geographical external validation cohort, and none in the temporal external validation cohort.

**Table 1:** Clinical characteristics of patients in the derivation, geographical, and temporal external validation cohorts

Characteristic	Derivation cohort (*n* = 789)	Geographical external validation cohort (*n* = 394)	Temporal external validation cohort (*n* = 185)
Age, years	45.0 (23.0)	44.0 (22.0)	47 (24.0)
Male, *n* (%)	567 (71.9)	290 (73.6)	126 (68.1)
Smoke, *n* (%)	181 (22.9)	95 (24.1)	51 (27.6)
Alcohol, *n* (%)	213 (27.0)	108 (27.4)	59 (31.9)
Diabetes, *n* (%)	53 (6.7)	29 (7.4)	18 (9.7)
Hypertension, *n* (%)	212 (26.9)	100 (25.4)	51(27.6)
HBV, *n* (%)	20 (2.5)	11 (2.8)	1 (0.5)
CHD, *n* (%)	20 (2.5)	9 (2.3)	6 (3.2)
Pre-op pneumocephalus, *n* (%)	43 (5.4)	4 (1.0)	1 (0.5)
Pre-op infection, *n* (%)	104 (13.2)	61 (15.5)	38 (20.5)
Pre-op seizures, *n* (%)	54 (6.8)	29 (7.4)	14 (7.6)
Pre-op fluid collections, *n* (%)	58 (7.4)	53 (13.5)	18 (9.7)
Pre-op hydrocephalus, *n* (%)	97 (12.3)	41 (10.4)	40 (21.6)
Skull defect area, cm^2^	117.0 (77.0)	104.0 (74.0)	104 (80.0)
Unilateral skull defect, *n* (%)	723 (91.6)	361 (91.6)	163 (88.1)
DC-CP interval, months	4.0 (3.0)	3.0 (3.0)	3 (2.0)
Pre-op V-P, *n* (%)	65 (8.2)	37 (9.4)	24 (13.0)
Surgery time, min	145.0 (95.0)	170.0 (120.0)	135 (90.0)
N-P drainage, *n* (%)	236 (29.9)	88 (22.3)	68 (36.8)
Materials			
Peek, *n* (%)	432 (54.8)	258 (65.5)	154 (83.2)
Composite material, *n* (%)	56 (7.1)	12 (3.0)	0 (0.0)
Titanium, *n* (%)	301(38.1)	124 (31.5)	31 (16.8)
GCS	15.0 (3.0)	15.0 (4.0)	15.0 (7.0)
GOS			
Death, *n* (%)	0 (0.0)	0 (0.0)	0 (0.0)
Vegetative state, *n* (%)	94 (11.9)	65 (16.5)	48 (25.9)
Severe disability, *n* (%)	136 (17.2)	63 (16.0)	67 (36.2)
Moderate disability, *n* (%)	140 (17.7)	69 (17.5)	29 (15.7)
Good recovery, *n* (%)	419 (53.1)	197 (50.0)	41 (22.2)
BI			
Complete dependency, *n* (%)	168 (21.3)	102 (25.9)	98 (53.0)
Moderate dependency, *n* (%)	62 (7.9)	26 (6.6)	17 (9.2)
Partial dependency, *n* (%)	140 (17.7)	70 (17.8)	30 (16.2)
Independent, *n* (%)	419 (53.1)	196 (49.7)	40 (21.6)
Craniectomy indication			
Trauma, *n* (%)	531 (67.3)	257 (65.2)	119 (64.3)
ICH, *n* (%)	207 (26.2)	101 (25.6)	51 (27.6)
Infraction, *n* (%)	26 (3.3)	15 (3.8)	7 (3.8)
Tumor, *n* (%)	25 (3.2)	21 (5.3)	8 (4.3)
Complications			
Infection, *n* (%)	22 (2.8)	10 (2.5)	5 (2.7)
Fluid collections, *n* (%)	111 (14.1)	83 (21.1)	40 (21.6)
Pneumocephalus, *n* (%)	22 (2.8)	10 (2.5)	10 (5.4)
Intracranial hemorrhage, *n* (%)	23 (2.9)	10 (2.5)	3 (1.6)
Hydrocephalus, *n* (%)	34 (4.3)	19 (4.8)	4 (2.2)
Seizures, *n* (%)	24 (3.0)	14 (3.6)	6 (3.2)
Overall complications, *n* (%)	205 (26.0)	115 (29.2)	51 (27.6)
Reoperations, *n* (%)	13 (1.6)	8 (2.0)	0 (0.0)

DC-CP interval, the time interval (in months) between decompressive craniectomy (DC) and cranioplasty (CP); GOS, Glasgow Outcome Scale; GCS, Glasgow Coma Scale; BI, Barthel Index, HBV, hepatitis B virus infection; CHD, coronary heart disease; N-P drainage, postoperative placement of subcutaneous negative-pressure drainage tubes; Pre-op V-P, preoperative ventriculoperitoneal shunt status; Pre-op, preoperative.

### Feature selection and model performance comparison

**Fig. 2:**
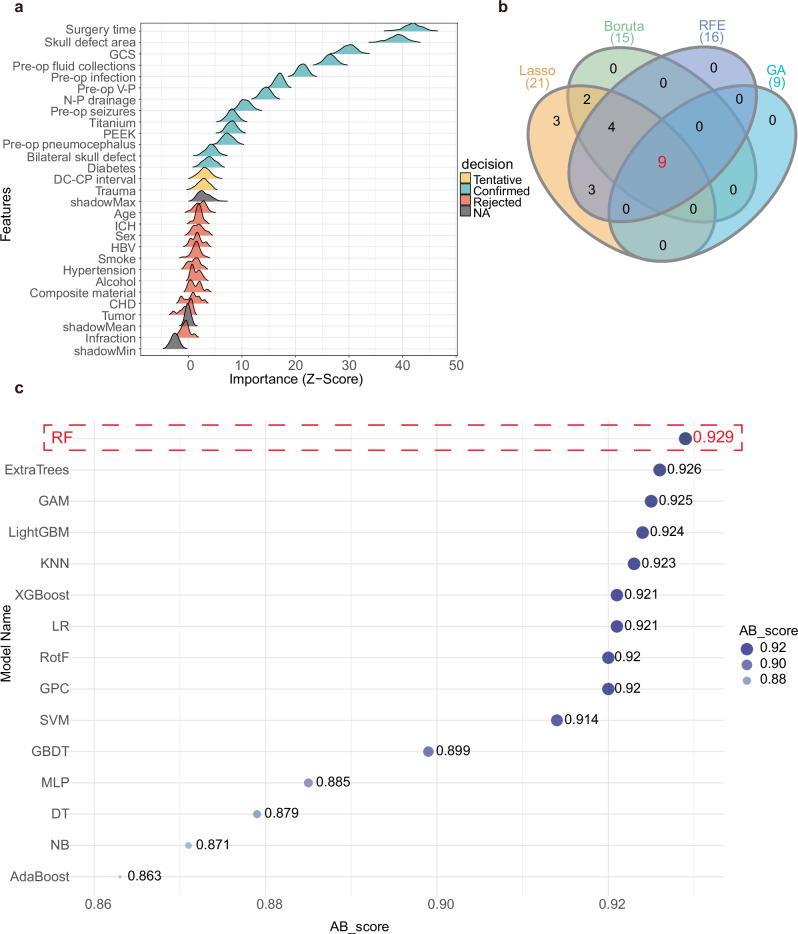
Feature selection and model performance comparison. **a** Feature importance ridge plot for variable selection based on Boruta. **b** Variable Venn diagram screened by four methods. **(c)** Bubble plots for comparing machine learning model performance based on the AB_score. DC-CP interval the time interval (in months) between decompressive craniectomy (DC) and cranioplasty (CP), GOS Glasgow Outcome Scale, GCS Glasgow Coma Scale, BI Barthel Index, HBV hepatitis B virus infection, CHD coronary heart disease, N-P drainage postoperative placement of subcutaneous negative-pressure drainage tubes, Pre-op V-P preoperative ventriculoperitoneal shunt status, Pre-op preoperative, GAM generalized additive model, LR logistic regression, GBDT gradient-boosted decision tree, KNN k-nearest neighbor, LightGBM light gradient boosting machine, RotF rotation forest, XGBoost extreme gradient boosting, NB naive Bayes, AdaBoost adaptive boosting, MLP multilayer perceptron, SVM support vector machine, DT decision tree, ExtraTrees extremely randomized trees, GPC Gaussian process classifier, RF random forest.

The feature selection process using the Boruta algorithm is illustrated in Fig. 2a. After 500 iterations, 13 of the 26 input features were confirmed as important, with 2 additional features marked as tentative. The selection procedures for LASSO, RF-RFE, and GA are presented in Fig. S6. A total of 9 overlapping predictors identified across all four methods were retained for model construction (Fig. 2b), including surgery time, skull defect area, Glasgow Coma Scale (GCS), preoperative fluid collections, preoperative infection, use of subcutaneous negative-pressure drainage, titanium mesh, preoperative ventriculoperitoneal (V-P) shunt, and the time interval between decompressive craniectomy and cranioplasty. The distributions of the nine selected predictors are shown in Fig. S7.

Based on the selected features, 15 ML models were developed to predict postoperative complications after cranioplasty. Figure 2c presents a comparison of the ${\rm{A}}{{\rm{B}}}_{{\rm{score}}}$ across different ML models. The Random Forest (RF) model achieved the highest ${\rm{A}}{{\rm{B}}}_{{\rm{score}}}$(0.929), outperforming all other algorithms and was selected as the final model for further evaluation. The optimal cutoff of the final RF model for predicting postoperative complications was 0.366 (Table S4). At this cutoff, the corresponding sensitivity, specificity, true/false positives (TP/FP), true/false negatives (TN/FN), positive predictive value (PPV), negative predictive value (NPV), and F1 score are reported in Table S5.

### Model evaluation and subgroup performance analysis

**Fig. 3:**
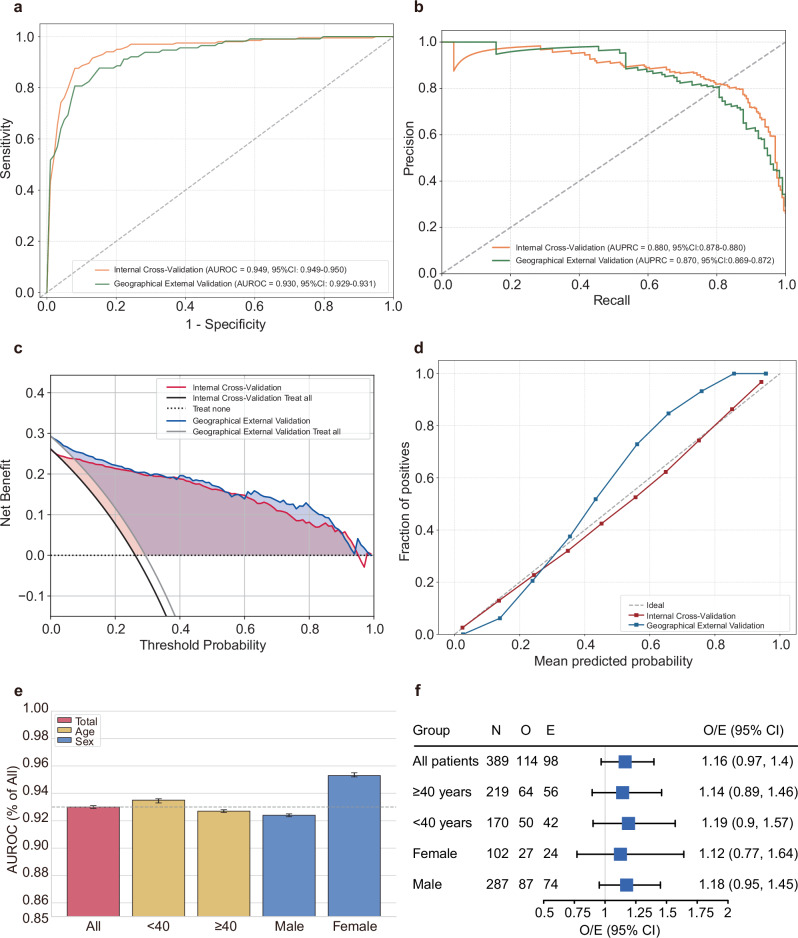
Model performance evaluation in the derivation cohort and geographical external validation cohort. **a** Receiver operating characteristic curves. **b** Precision-recall curves. **c** Decision curve analysis. **d** Calibration curves. **e** Discrimination performance across subgroups in the geographical external validation cohort. The dotted line represents the mean AUROC of the overall cohort. **f** Calibration performance across subgroups in the geographical external validation cohort. AUROC area under the receiver operating characteristic curve, AUPRC area under the precision–recall curve. O observed. E expected.

We comprehensively evaluated the final RF model in terms of both discrimination and calibration. The AUROC was 0.949 (95% CI: 0.949–0.950) in the internal cross-validation, and 0.930 (95% CI: 0.929–0.931) in the graphical external validation cohort (Fig. 3a). Consistent with AUROC, the area under the precision-recall curve (AUPRC) remained high, with values of 0.880 (95% CI: 0.878–0.880) and 0.870 (95% CI: 0.869–0.872), respectively (Fig. 3b).

Clinical utility was evaluated using DCA (Fig. 3c). Across a wide range of threshold probabilities, the model consistently provided a greater net benefit than both the treat-all and treat-none strategies. Calibration performance is shown in Fig. 3d. The predicted probabilities demonstrated overall good agreement with the observed outcomes. Slight overestimation was noted at higher predicted risk levels in the geographical external validation cohort.

To further understand the predictive performance of the final model in specific patient populations, subgroup analyses were conducted based on age (<40 and ≥40 years) and sex (male, female) in the geographical external validation cohort. Although the AUROC was slightly reduced in ≥40 years patients and male patients compared with the overall cohort, their AUROCs were still higher than 0.92 (Fig. 3e). Calibration in subgroups was evaluated using a forest plot of observed-to-expected (O/E) ratios (Fig. 3f). The overall value was 1.16 (95% CI:0.97–1.40) and there was no significant prediction bias compared with the true outcome in all subgroups.

### Complication-specific model development

Given that individual complications may involve distinct mechanisms and require personalized management, we further constructed dedicated ML models for major complications, including infection, pneumocephalus, fluid collection, hydrocephalus, seizures, intracranial hemorrhage, and reoperations. Final model selection was guided by a comprehensive assessment of $A{B}_{\mathrm{score}}$, calibration, and clinical utility. The optimal models were rotation forest (RotF) for infection and pneumocephalus; RF for fluid collection and reoperations; generalized additive model (GAM) for hydrocephalus; and logistic regression for seizures and intracranial hemorrhage. Performance metrics are summarized in Table S6, with calibration curves, decision curve analyses, and SHapley Additive exPlanations (SHAP) summary plots presented in Figs. S8–S10.

### Temporal external validation of the models

**Fig. 4:**
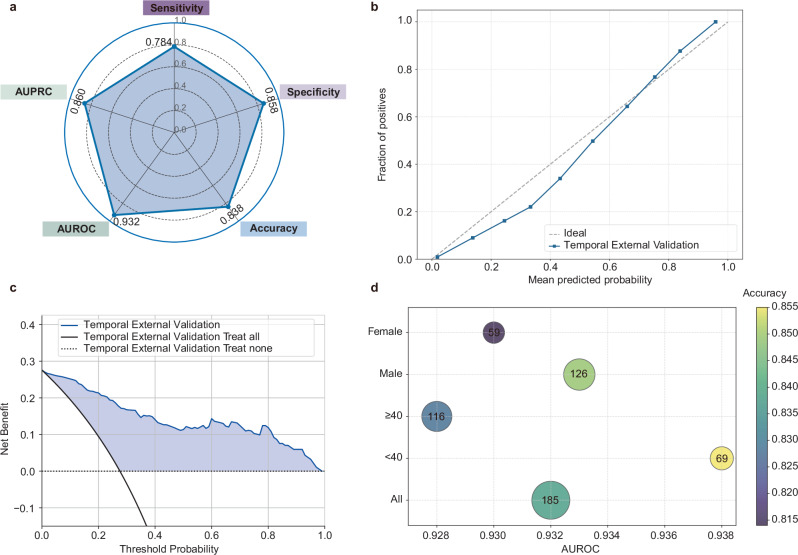
Temporal external validation of the final model. **a** Radar plot of five key evaluation metrics. **b** Calibration curves. **c** Decision curve analysis. **d** Bubble chart of subgroup performance. The *X*-axis indicates the area under the receiver operating characteristic curve (AUROC), while the *Y*-axis represents different subgroups (age and sex). The size of each bubble reflects the sample size, and the color gradient represents the prediction accuracy, with lighter colors indicating higher accuracy. Numerical labels within the bubbles denote the sample size of each subgroup. AUROC area under the receiver operating characteristic curve, AUPRC,area under the precision–recall curve.

We further evaluated the temporal generalizability of both the overall complication model and the complication-specific models using an independent temporal validation cohort. The final overall complication model achieved an AUROC of 0.932 and an overall accuracy of 0.838 (Fig. 4a). Calibration analysis (Fig. 4b) showed good agreement between predicted and observed risks, with the calibration curve closely following the ideal diagonal line. Decision curve analysis (Fig. 4c) demonstrated net clinical benefit across a wide range of threshold probabilities. Subgroup analysis (Fig. 4d) further demonstrated the model’s stable performance across age and sex subgroups, with AUROC values exceeding 0.9 and accuracy exceeding 0.8 in all subgroups.

Performance metrics for complication-specific models are provided in Table S7. AUROC values were consistently high across all outcomes (0.851–0.986), indicating strong overall discrimination. However, for intracranial hemorrhage, hydrocephalus, and seizures, the models showed comparatively lower AUPRC values (0.536, 0.574, and 0.600, respectively) and a higher proportion of false-positive predictions.

### Model interpretability and feature interaction analysis of the overall complication model

**Fig. 5:**
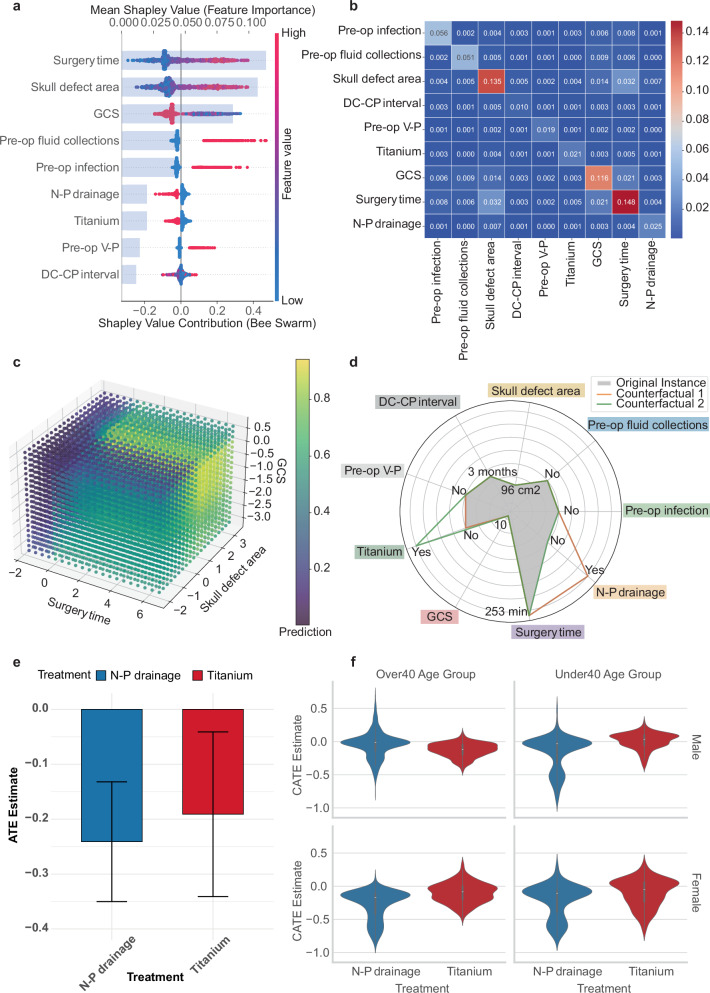
Model interpretability and causal inference analysis. **a** SHAP summary plot (bee swarm). This plot evaluates the contribution of each feature to the model using mean SHAP values, displayed in descending order, with color indicating feature value (red = high, blue = low). **b** SHAP interaction heatmap. This plot quantifies pairwise feature interactions, where darker colors represent stronger interaction effects. **c** Three-dimensional partial dependence plot (3D PDP). This plot visualizes the joint effect of surgery time, GCS and skull defect area on the predicted probability. Color intensity ranging from dark to light represents the predicted probability of postoperative complications, with lighter colors indicating higher risk. **d** Radar plot of counterfactual explanations. This plot presents two counterfactual scenarios generated by DiCE. **e** Bar plot of ATE estimates. The plot shows the estimated ATEs for two modifiable surgical variables: N–P drainage and use of titanium mesh as the cranioplasty material. **f** Violin plot of CATE estimates. The plot presents subgroup-level CATE estimates for N–P drainage and use of titanium mesh as the cranioplasty material, stratified by age and sex. DC-CP interval the time interval (in months) between decompressive craniectomy (DC) and cranioplasty (CP), GCS Glasgow Coma Scale, BI Barthel Index, N-P drainage postoperative placement of subcutaneous negative-pressure drainage tubes, Pre-op V-P preoperative ventriculoperitoneal shunt status, Pre-op preoperative, DiCE Diverse Counterfactual Explanations, ATE average treatment effect, CATE conditional average treatment effect.

We employed SHAP to assess feature contributions at both the global and individual levels. Global feature importance is shown in Fig. 5a, where contributions were quantified using mean SHAP values and ranked in descending order. Surgery time, skull defect area, and GCS were identified as the top three predictors. Local explanations were further used to illustrate how individual predictions were generated based on patient-specific feature values. Corresponding visualizations, including force plots, decision plots, and waterfall plots, are presented in Figs. S11 and S12.

To further explore potential interactions among features, we generated a SHAP interaction heatmap (Fig. 5b). Skull defect area, GCS, and surgery time exhibited high self-interaction SHAP values. Their marginal effects on the predicted risk of postoperative complications were examined using one-dimensional (1D) partial dependence plots (PDPs) (Fig. S13). And their joint effects were visualized using a three-dimensional (3D) PDP (Fig. 5c). The plot showed that low GCS and a larger skull defect area were associated with a higher predicted risk of postoperative complications. This effect was more pronounced in patients with longer surgery time than in those with shorter surgery time.

### Counterfactual analysis and causal inference of the modifiable surgical variables

Identifying modifiable surgical variables is of particular importance in neurosurgical practice. In our model, the use of subcutaneous negative-pressure (N-P) drainage and titanium mesh in cranioplasty was associated with a lower predicted risk of postoperative complications (Fig. 5a).

To examine whether changes to these factors could influence model predictions, we performed counterfactual analysis using the Diverse Counterfactual Explanations (DiCE) method. The results showed that modifying either the drainage method or the cranioplasty material alone was sufficient to convert a high-risk prediction into a low-risk outcome in selected patients (Fig. 5d).

To further validate these findings, causal effects of these modifiable surgical factors on postoperative complication risk were estimated using Double Machine Learning (DML). Both N-P drainage and titanium mesh were associated with reduced predicted complication risk, with average treatment effects (ATEs) of −0.241 (95% CI: −0.35 to −0.132) and −0.191 (95% CI: −0.341 to −0.041), respectively (Fig. 5e). Subgroup-specific conditional average treatment effects (CATEs) were subsequently estimated to assess heterogeneity in treatment response (Fig. 5f). The protective effects of titanium mesh and N-P drainage were observed in most age and sex subgroups. However, among males over 40 years, the estimated CATE for N-P drainage exceeded zero (CATE = 0.009), indicating no protective benefit in this subgroup. Detailed CATE results are provided in Table S9.

Finally, sensitivity analysis was conducted to assess the robustness of the estimated ATEs and CATEs. Random noise variables were introduced into the estimation process, and treatment effects were re-estimated. No statistically significant differences were observed between the original and perturbed estimates (all *p* > 0.05; Table S8, S9).

### Accessible web application for clinical utility

The overall and complication-specific models were integrated into a web-based application with eight prediction modules (Fig. S14). Users can input the required feature values under the relevant module, and the application will automatically calculate and display the predicted risk for the selected complication (Fig. S15). The web application can be accessed online at the following link: http://www.cranioplastycomplicationprediction.top/.

In addition, we translated the core methodologies of this study into a generalizable methodological framework platform (Fig. S16; https://surgical-complication-risk-prediction.streamlit.app/). This platform provides a reproducible pipeline for predicting postoperative complications across diverse surgical procedures.

## Discussion

ML techniques have been widely used in modern medical research due to their ability to process high-dimensional data and capture complex, nonlinear interactions among variables^12^. They have demonstrated strong predictive performance in multiple clinically challenging domains, including acute critical illness risk stratification, postoperative functional outcome prediction, and in-hospital mortality prediction^13–15^. However, their application in cranioplasty remains limited. To our knowledge, this is the first study to systematically compare 15 machine learning algorithms for predicting postoperative complications following cranioplasty, based on large-scale multicenter data.

Individualized assessment of postoperative complication risk represents an important component of perioperative care in patients undergoing cranioplasty. By identifying patients at elevated risk, the proposed model may support more targeted postoperative management strategies. For example, patients predicted to be at increased risk of postoperative seizures may benefit from closer neurophysiological monitoring or consideration of prophylactic antiepileptic therapy. Similarly, for patients at increased risk of postoperative fluid collection, drainage strategies such as prolonging drainage duration may be adjusted to reduce the likelihood of this complication.

As highlighted by Thomas H. Shin et al.^16^, the goal of ML in surgical outcomes research is not simply to improve risk prediction, but to identify underrecognized modifiable risk factors. For neurosurgeons, a critical question is whether optimizing surgical strategies can effectively reduce the risk of complications following cranioplasty. In this context, causal machine learning offers a potential solution.

Unlike traditional ML, which identifies high-risk patients without informing specific actions, Causal ML aims to answer “what if” questions and quantify the effects of potential interventions using data from randomized controlled trials (RCTs) and real-world sources such as clinical registries and EMRs^17^. By using DML and the T-learner framework, we found that both N-P drainage and the use of titanium mesh exhibited protective effects against postoperative complications following cranioplasty. Notably, N-P drainage is a novel and modifiable surgical factor that has not been previously reported in literature. The continuous evacuation of postoperative blood and exudate may reduce the risk of hematoma or fluid collection, thereby contributing to its protective effect.

Furthermore, the choice of cranioplasty material, particularly the use of titanium mesh, warrants further discussion in the context of existing evidence. Rosenthal et al.^18^ reported that complication rates with polyetheretherketone (PEEK) implants are comparable to those of other materials. In contrast, Rosinski et al.^19^ found higher infection rates in patients with PEEK custom implants than in those with titanium meshes. However, the limited sample sizes of these studies weaken their conclusions. Using a large multicenter dataset and causal inference methods, we found that the use of titanium mesh as a cranioplasty material was associated with a lower risk of overall postoperative complications. The lower complication rate associated with titanium mesh may be attributed to its superior biocompatibility and inherent antibacterial properties^20^, which help reduce the risk of infection and inflammation. In addition, it offers good intraoperative malleability, allowing for manual trimming and contouring to achieve better conformity to the defect site and enhanced implant stability. This adaptability may help reduce dead space and consequently lower the risk of postoperative fluid collection.

In practical terms, this protective association is particularly relevant for patients with impaired neurological status before surgery, as minimizing postoperative complications is essential in preventing further deterioration. In such scenarios, when synthetic materials such as PEEK, titanium mesh, and polymethyl methacrylate–hydroxyapatite composite cement are all viable options, neurosurgeons may consider titanium mesh as a more favorable choice.

Several previous studies have also attempted to develop prediction models in the context of cranioplasty. Kimchi, G et al.^21^ used survival analysis to predict the probability of post-cranioplasty infection and identified preoperative neurological disability as the strongest predictor. Klieverik, V.M et al.^22^ developed a Cox-based model to predict cranioplasty implant survival and reported several clinical determinants. Lu, Y et al.^23^ proposed a logistic-regression model incorporating modified brain-collapse ratio with comorbidity burden to predict postoperative complications. These earlier studies highlight growing interest in predictive modeling for cranioplasty. However, they were constrained by small single-center samples, modest predictive performance, reliance on traditional regression methods, and the absence of external validation, limiting their clinical applicability. In contrast, our study addressed these limitations by leveraging a large multicenter dataset, systematically comparing multiple machine-learning algorithms, and validating the final model across both geographical and temporal cohorts. Moreover, by applying causal ML methods, we were able to identify potentially modifiable surgical variables, thereby providing guidance for surgical decision-making.

Composite outcomes are commonly used in surgical outcomes research to capture the overall clinical impact of heterogeneous postoperative complications. In the context of cranioplasty, overall complication outcomes can inform perioperative management, guide postoperative monitoring strategies, and reflect overall patient outcomes. Accordingly, prior studies have frequently adopted an overall complication endpoint to investigate risk factors associated with postoperative complications^9,10^. This analytical paradigm is not unique to cranioplasty and has been widely applied across postoperative complication and adverse event research. For instance, Chen et al.^24^ combined multiple postoperative pulmonary complications into a composite endpoint to develop a risk prediction model. Similarly, Mahajan et al.^25^ defined major adverse cardiac and cerebrovascular events as a composite outcome comprising postoperative type I or II myocardial infarction, cardiogenic shock or acute heart failure, unstable angina, and stroke, and subsequently constructed predictive models based on this composite endpoint.

To further enhance clinical relevance, we also developed separate models targeting individual complications. In clinical practice, the overall model serves as an initial screening tool to identify patients at higher postoperative risk who may require intensified monitoring, while the specific models further identify the most likely complication types to guide targeted preventive and management strategies. Together, these complementary models support comprehensive postoperative risk assessment and individualized perioperative care.

Despite the promising results of this study, several limitations warrant consideration. First, our models were developed using data from Chinese patients. Future studies are needed to evaluate their generalizability beyond China. Second, although the models effectively predicted the occurrence of postoperative complications following cranioplasty, they could not determine the timing of these events. Future research should focus on integrating temporal analyses to predict the timing of complications, which could further enhance clinical utility. Third, our study only focused on in-hospital complications following cranioplasty and excluded post-discharge events due to inconsistent and incomplete post-discharge data across centers. Fourth, although the models demonstrated robust sensitivity and strong discrimination overall, the complication-specific models for intracranial hemorrhage, seizures, and hydrocephalus showed a higher proportion of false-positive predictions in the temporal external validation cohort. Fifth, our analysis did not include autologous bone as its use was rare across the participating centers. The infrequent use of autologous bone at our participating centers makes it difficult to incorporate this surgical strategy into meaningful statistical analyses. Future studies with larger multicenter datasets may enable more robust evaluation of autologous bone and its comparative impact on postoperative outcomes.

In conclusion, our study successfully developed the first interpretable ML-based clinical tool for predicting postoperative complications after cranioplasty using preoperative and intraoperative data extracted from EMRs. With its high predictive accuracy and practical accessibility, this non-invasive tool has the potential to enhance perioperative risk stratification by shifting complication prediction from experience-based judgment to a data-driven approach, ultimately improving patient outcomes in neurosurgical practice. Further research is warranted to validate the real-world applicability of our clinical tool across diverse healthcare settings.

## Methods

### Ethics statement

This study was conducted in accordance with the Declaration of Helsinki and approved by the Institutional Review Boards of Qilu Hospital of Shandong University (KYLL-202407-041), Daping Hospital of Army Medical University [(2024) 293], and Tang-Du Hospital (K202411-26). As a retrospective study, the requirement for informed consent was waived. The study is registered with ClinicalTrials.gov (NCT06740773, registered on December 18, 2024) and conducted in accordance with the Transparent Reporting of a Multivariable Prediction Model for Individual Prognosis or Diagnosis with Artificial Intelligence (TRIPOD + AI) guidelines^26^. Patients or members of the public were not involved in the design, conduct, reporting, interpretation, or dissemination of this study.

### Study population

This multicenter retrospective cohort study included patients of all ages who underwent cranioplasty in the neurosurgery departments of three independent hospitals. The derivation cohort consisted of patients who underwent cranioplasty at two independent tertiary hospitals (Qilu Hospital of Shandong University and Daping Hospital of Army Medical University) between January 1, 2015, and July 31, 2023. This cohort was used for model training and internal validation through 5-fold cross-validation. The geographical external validation cohort included patients who underwent cranioplasty at another independent tertiary hospital (Tangdu Hospital of Air Force Medical University) during the same period. The temporal external validation cohort included patients who underwent cranioplasty at Qilu Hospital of Shandong University between August 1, 2023, and January 1, 2025. Individuals with a history of prior cranioplasty, severe comorbidities (including significant cardiac, liver, kidney, or immune system dysfunction), congenital cranial defects, or substantial missing data were excluded from the study. The primary endpoint of this study was the occurrence of in-hospital complications following cranioplasty.

### Sample size estimation

To determine the minimum sample size required for developing the clinical prediction models, we followed the four-step approach, using pmsampsize package in R and the web tool BeyondEPV (https://mvansmeden.shinyapps.io/BeyondEPV)^27^. The following parameters were prespecified: number of predictor parameters = 9, shrinkage factor = 0.9, outcome prevalence = 0.2, minimum acceptable C-statistic = 0.8, and mean absolute percentage error (MAPE) = 0.05. Based on these criteria, the minimum required sample size was estimated to be 400.

### Data collection and quality control

Both the factors influencing postoperative complications following cranioplasty and the specific outcome variables for complications were identified through a comprehensive literature review, including systematic reviews, meta-analyses, primary studies, and expert clinical opinions^28–30^. Postoperative complications were classified into six major categories: infection, intracranial hemorrhage, fluid collections, hydrocephalus, seizures, and pneumocephalus. Only complications requiring active clinical intervention were counted as clinically significant complications in this study. Minor or self-limiting abnormalities were not considered as complications. In addition, reoperation, defined as a return to the operating room for removal of the implanted material due to severe complications, was also recorded as a separate outcome. Data was extracted from patients’ EMRs. Detailed information about factors and complications is provided in Table S1.

To ensure data consistency and reliability, a standardized multicenter database was established. Data collectors were uniformly trained, and clinical information was recorded using standardized forms. Following data collection, a cross-center quality control process was implemented. A random 30% sample of records from each center was independently reviewed by neurosurgeons from other participating centers. A minimum data accuracy rate of ≥90% was required; centers not meeting this threshold were mandated to re-evaluate and correct their data.

### Data preprocessing

To handle missing data, the extent and pattern of missingness across all variables were first assessed (Fig. S1). The missingness mechanism for the skull defect area variable was first assessed by clinical experts and then verified statistically, supporting a Missing Completely at Random assumption (Table S2)^31,32^. Six commonly used imputation methods were subsequently evaluated using cross-validation, with the normalized root mean square error (NRMSE) as the primary evaluation metric. Among these, missForest^33^ was ultimately chosen for subsequent analyses (Fig. S2) due to its superior performance in minimizing NRMSE. A sensitivity analysis was conducted to verify that the imputation process preserved data distribution consistency and introduced no bias, as expected under the assumption (Fig. S3). Outliers were identified using a four-layer autoencoder trained with the Adam optimizer and mean squared error loss. Samples with reconstruction errors exceeding the 2σ threshold were considered outliers and excluded from further analysis^34^ (Fig. S4). The impact of this filtering step on model performance was evaluated through a sensitivity analysis, as detailed in Table S3. Continuous variables were standardized using Z-score normalization, and categorical variables were standardized through one-hot encoding^35^. To avoid data leakage, all preprocessing steps were independently applied to the derivation and geographical external validation cohorts. The temporal external validation dataset was left unprocessed to reflect real-world deployment conditions.

### Feature selection

To address multicollinearity arising from highly correlated features, correlation filtering was applied to remove redundancy, ensuring that all pairwise correlation coefficients fell below 0.6. The variance inflation factor was subsequently calculated to confirm the absence of multicollinearity among the remaining variables (Fig. S5). Feature selection was performed using four distinct algorithms: Boruta^36^, Lasso^37^, Random Forest–based Recursive Feature Elimination (RF-RFE)^38^, and Genetic Algorithm (GA)^39,40^ (Fig. S6). The final feature set was determined by identifying the intersection of variables selected by all four methods and was visualized using a Venn diagram^41^. Experienced neurosurgeons from three centers then reviewed the selected features and finalized the predictors, ensuring high face validity and ease of implementation.

### Model development, comparisons, and evaluation

Fifteen ML models were developed to predict the risk of complications after cranioplasty: GAM, logistic regression, gradient-boosted decision tree, K-nearest neighbor, light gradient boosting machine, RotF, extreme gradient boosting, naive Bayes, adaptive boosting (AdaBoost), multilayer perceptron, support vector machine, decision tree, extremely randomized trees (ExtraTrees), Gaussian process classifier and random forest (RF). To minimize overfitting and enhance model robustness before external validation, 5-fold cross-validation was performed on the derivation cohort. The optimal cutoff for model was determined by maximizing the Youden index (sensitivity + specificity − 1). The 95% confidence interval was estimated using the bias-corrected and accelerated (BCA) bootstrap method^42^.

To select the optimal prediction models for each complication, we defined a novel metric, ${\mathrm{AB}}_{\mathrm{score}}$, which combines the Area Under the Receiver Operating Characteristic Curve (AUROC) and the Brier score to assess both the discriminative and calibration abilities of each model. To avoid overly optimistic estimates derived from the training data, performance metrics from the training cohort were not used for model evaluation. Only internal cross-validation and external validation metrics were considered to reliably assess the generalizability of each model.

The mathematical formula for $A{B}_{{score}}$ is presented in Eq. (1)

1 $$A{B}_{score}=\alpha * \overline{AUROC}+\alpha * (1-\overline{Briers\,core})$$

2 $$\alpha =0.5$$

where $\overline{AUROC}$ and $\overline{Briers\,core}$ represents the arithmetic mean of the AUROC values and Brier score from the internal cross-validation and geographical external validation sets, respectively. $\alpha$ is a weight factor, and by setting $\alpha =0.5$, equal emphasis is placed on discrimination and calibration in the $A{B}_{\mathrm{score}}$. $1-\overline{Briers\,core}$ is used to align with the positive direction of AUROC.

The discrimination ability of the final model was assessed using the receiver operating characteristic (ROC) curve and Precision–Recall (PR) Curve, while calibration was evaluated with the Brier score and calibration curve^43^. In addition, decision curve analysis (DCA) was used to evaluate the net benefit of the model at different thresholds^44^. Furthermore, to evaluate model fairness, we assessed the model’s performance across demographic subgroups, including age (<40 and ≥40 years) and sex (male, female), within the external validation cohort^45^.

### Complication-specific modeling

Separate machine learning models were developed for each major type of postoperative complication. To address severe class imbalance in complication-specific modeling, ADASYN^46^ was applied to the data to augment minority-class samples and improve model sensitivity for rare outcomes.

### Model explanation and causal inference analysi*s*

To address the inherent opacity of machine learning models, SHAP, a game-theoretic approach, was employed to quantify the contribution of individual features to deviations from the mean prediction using SHAP values^47^. PDPs were additionally applied to visualize interactions among key SHAP-identified features and their combined effects on model predictions^48,49^.

To investigate the potential causal effects of modifiable surgical factors on postoperative complications, a two-step approach was employed. First, DiCE^50^ was used to simulate hypothetical adjustments in clinically actionable variables and to explore whether such changes could influence model-predicted complication risks. Subsequently, causal inference methods were applied to quantify the effects of these variables. DML was used to estimate ATEs^51^, while a T-learner framework was employed to evaluate CATEs across patient subgroups^52^. Sensitivity analyses were conducted to assess the robustness of the causal estimates.

### Statistical analysis

Patient data were categorized into continuous and categorical variables. The normality of continuous variables was assessed using the Kolmogorov–Smirnov test. Variables with a normal distribution were described as mean ± standard deviation, while those with a skewed distribution were presented as median with interquartile range. Categorical variables were reported as counts and percentages. Data analyses were performed using IBM SPSS Statistics for Windows (IBM Corp., released 2019, version 26.0), Python (version 3.8.2), and R (version 4.2.2).

## Supplementary information

Supplementary Information

## Data Availability

The datasets analyzed implemented during this study are available from the corresponding author upon reasonable request. The codes are uploaded on Github. (GitHub - BigEarAsk/A-Causal-and-Interpretable-Machine-Learning-Framework-for-Post-Cranioplasty-Complications: Code for training and validation).
