# Supplementary material for: A Causal and interpretable machine learning framework for postcranioplasty risk prediction and surgical decision support
Source: NPJ Digit Med. 2026 Jan 21;9:184. doi: 10.1038/s41746-026-02370-6 (PMC12923646; doi:10.1038/s41746-026-02370-6)
Supplement: Supplementary file 1 — Supplementary Information [file 41746_2026_2370_MOESM1_ESM.pdf]

Supplementary Materials: A Causal and Interpretable Machine Learning Framework for Postcranioplasty Risk Prediction and Surgical Decision Support

Contents

Supplementary Tables..3

Table S1. Detailed information about study predictors and outcome variable....................3

Table S2. logistic regression for testing MCAR assumption of skull-defect area....................5

Table S3. Sensitivity analysis of model performance with and without autoencoder-based outlier removal....................6

Table S4. Youden Index of 15 ML models....................7

Table S5. Predictive performance of ML models in predicting overall complication....................8

Table S6. Predictive performance of ML models in predicting specific complications....................9

Table S7. Predictive Performance of Machine Learning Models for Specific Complications in the Temporal External Validation Cohort....................11

Table S8. Estimated Average Treatment Effects (ATE) of Surgical Variables with Robustness Checks....................11

Table S9. Estimated Conditional Average Treatment Effect (CATE) of Surgical Variables with Robustness Checks....................11

Supplementary Figures..13

Figure S1. Proportion of missing values across variables in the derivation cohort and external validation cohort. 13

Figure S2. NRMSE values of each imputation method 14

Figure S3. Sensitivity analysis of imputation results 15

Figure S4. Workflow of the designed autoencoder for outlier detection 16

Figure S5. Correlation filtering of highly correlated variables 17

Figure S6. Variable selection using Least Absolute Shrinkage and Selection Operator (LASSO), Recursive Feature Elimination (RFE), and genetic algorithm (GA) 19

Figure S7. Distribution of the Nine Selected Predictors Stratified by Postoperative Complication Status....................20

Figure S8. Calibration curves of the best-performing machine learning models for predicting each complication....................21

Figure S9. Decision curves of the best-performing machine learning models for predicting each complication....................22

Figure S10. SHAP summary plots of the best-performing machine learning models for predicting each complication. 23

Figure S11. Local SHAP explanations for representative samples 24
Figure S12. Local SHAP explanations by the SHAP method. 25
Figure S13. SHAP dependence plots of key predictive features 26
Figure S14. Flow chart of web deployment. 27
Figure S15. Web-based clinical decision-support platforms for postoperative complication risk prediction following cranioplasty 28
Figure S16. Generalizable methodological framework platform integrating model evaluation, interpretability, counterfactual, and causal inference analyses. 29

Supplementary Tables

Table S1. Detailed information about study predictors and outcome variable

Name	Description
Study Predictors	
Age	The age of the patient (in years)
Sex	The sex of the patient (Male/Female)
Smoke	A binary variable indicating whether the patient has a history of smoking
Alcohol	A binary variable indicating whether the patient has a history of alcohol consumption
Diabetes	Binary variable indicating whether the patient has a current diagnosis of diabetes mellitus.
Hypertension	Binary variable indicating whether the patient has a current diagnosis of hypertension
HBV	Binary variable indicating whether the patient has a current diagnosis of chronic hepatitis B infection
CHD	Binary variable indicating whether the patient has a current diagnosis of coronary heart disease
Skull defect area	Continuous variable measuring the area of the skull defect (square centimeters)
Skull defect side	Categorical variable indicating the location of the skull defect (Unilateral/Bilateral)
DC-CP interval	Continuous variable representing the time interval (in months) between decompressive craniectomy (DC) and cranioplasty (CP)
Craniectomy indication	Categorical variable indicating the primary reason for performing craniectomy, including tumor, infarction, intracerebral hemorrhage, and trauma
Pre-op V-P	Binary variable indicating whether the patient has a ventriculoperitoneal shunt placed prior to surgery
Materials	Categorical variable indicating the material used for cranioplasty including titanium mesh, polymethyl methacrylate-hydroxyapatite composite cement (Composite materials), polyetheretherketone (PEEK)
Surgery time	Continuous variable indicating the duration of the surgery (minutes)
N-P drainage	Binary variable indicating whether subcutaneous negative pressure drainage was used after surgery

GOS	Glasgow Outcome Scale, ranging from 1 to 5. Ordinal variable measuring functional outcome on a 5-point scale, with higher scores indicating better recovery
GCS	Glasgow Coma Scale (GCS), ranging from 3 to 15. Ordinal variable assessing the level of consciousness of the patient, with higher scores indicating better neurological function preoperatively
BI	Barthel Index, categorized into four levels. Ordinal variable assessing activities of daily living (ADL) independence (complete dependency/ moderate dependency/ partial dependency/ independent)
Pre-op pneumocephalus	Binary variable indicating whether preoperative CT imaging showed the presence of intracranial collection of air
Pre-op infection	Binary variable indicating whether the patient exhibited clinical signs of infection prior to surgery. Clinical signs may include symptoms such as cough, sputum production, chest pain, dyspnea, fever, chills, and fatigue. Diagnosis may also be supported by imaging findings from chest X-rays, CT scans, or other radiological assessments
Pre-op seizures	Binary variable indicating whether the patient had a history of seizures or experienced seizures prior to surgery
Pre-op fluid collections	Binary variable indicating whether preoperative CT imaging showed the presence of fluid collections in the subdural or epidural spaces
Pre-op hydrocephalus	Binary variable indicating whether preoperative CT imaging showed excessive cerebrospinal fluid (CSF) accumulation, resulting in ventricular enlargement or subarachnoid space widening
Outcome Variable	
Pneumocephalus	Binary outcome variable defined as newly developed or significantly increased intracranial air accumulation, identified by postoperative imaging compared to the preoperative baseline, and accompanied by relevant clinical symptoms. Only new or progressive postoperative pneumocephalus was classified as a complication. Cases where intracranial air remained stable in volume and distribution compared to preoperative imaging were not considered outcome events
Infection	Binary outcome variable defined as a new-onset intracranial or surgical site infection occurring during the postoperative course. Diagnosis was based on clinical symptoms (e.g., fever, wound redness/swelling, neurological deterioration), laboratory findings, and/or radiological evidence
Seizures	Binary outcome variable defined as the occurrence of new-onset seizures during the postoperative period, confirmed by clinical documentation or EEG findings
Fluid collections	Binary outcome variable defined as newly developed or significantly increased subcutaneous, subdural, or epidural fluid collections, identified by postoperative imaging compared to the preoperative baseline, and accompanied by relevant clinical symptoms. Only new or progressive postoperative fluid collections were classified as complications. Cases where fluid accumulation remained stable in volume and distribution compared to preoperative imaging were not considered outcome events
Hydrocephalus	Binary outcome variable defined as newly developed or significantly worsened ventricular enlargement, identified by postoperative imaging compared to the preoperative baseline, and accompanied by relevant clinical symptoms. Only new or progressive postoperative hydrocephalus was classified as a complication. Cases where ventricular size remained stable compared to preoperative imaging were not considered outcome events
Intracranial hemorrhage	Binary variable indicating whether intracerebral hemorrhage, intraventricular hemorrhage, subarachnoid hemorrhage, subdural hematoma, or epidural hematoma occurred postoperatively
Reoperations	Binary variable indicating whether the patient required additional surgeries due to severe complications
Overall complications	Binary variable indicating whether any complications mentioned above occurred postoperatively

All outcomes and predictor data were extracted retrospectively from the electronic medical record system. The original documentation was completed by board-certified attending physicians, each with over 15 years of clinical experience in neurosurgery. As all variables were recorded during routine clinical care, no post hoc subjective

interpretation was required by the study investigators.

Table S2. logistic regression for testing MCAR assumption of skull-defect area

**Predictor variable**	**Type**	**Comparison**	***p**** **(Derivation cohort)**	***p**** **(External Validation cohort)**
Surgery time	Continuous		0.091	1.000
Materials	Categorical	PEEK vs Composite material	0.676	0.382
N-P drainage	Binary	Yes vs No	0.702	1.000
Hypertension	Binary	Yes vs No	0.859	1.000
Alcohol	Binary	Yes vs No	0.903	1.000
BI	Ordinal		1.000	1.000
Skull defect side	Binary	Unilateral vs Bilateral	1.000	0.623
Pre-op hydrocephalus	Binary	Yes vs No	1.000	1.000
Diabetes	Binary	Yes vs No	1.000	1.000
GOS	Ordinal		1.000	0.702
Pre-op V-P	Binary	Yes vs No	1.000	1.000
GCS	Ordinal		1.000	1.000
Materials	Categorical	PEEK vs Titanium mesh	1.000	1.000
Pre-op seizures	Binary	Yes vs No	1.000	1.000
Age	Continuous		1.000	1.000
Pre-op pneumocephalus	Binary	Yes vs No	1.000	1.000
Smoke	Binary	Yes vs No	1.000	1.000
Sex	Binary	Female vs male	1.000	1.000
Craniectomy indication	Categorical	Trauma vs Infraction	1.000	1.000
Craniectomy indication	Categorical	Trauma vs ICH	1.000	1.000

Pre-op fluid collections	Binary	Yes vs No	1.000	1.000
DC-CP interval	Continuous		1.000	1.000
HBV	Binary	Yes vs No	1.000	1.000
CHD	Binary	Yes vs No	1.000	1.000
Craniectomy indication	Categorical	Trauma vs Tumor	1.000	1.000
Pre-op infection	Binary	Yes vs No	1.000	1.000

Abbreviations: p^*: Bonferroni-adjusted p-value. DC-CP interval, the time interval (in months) between decompressive craniectomy (DC) and cranioplasty (CP); GOS, Glasgow Outcome Scale; GCS, Glasgow Coma Scale; BI, Barthel Index; HBV, hepatitis B virus infection; CHD, coronary heart disease; N-P drainage, postoperative placement of subcutaneous negative-pressure drainage tubes; Pre-op V-P, preoperative ventriculoperitoneal shunt status; Pre-op, preoperative.

Note: Each observed covariate was entered separately into a univariable logistic regression model, with the missingness indicator (1 = missing, 0 = observed) for the variable skull defect area as the dependent variable. The analyses were performed independently in both the derivation and external validation cohorts to examine whether the probability of missingness was associated with observed variables.

Table S3. Sensitivity analysis of model performance with and without autoencoder-based outlier removal

Name	Accuracy	AUROC	Sensitivity	Specificity	PPV	NPV	F1	Brier score
Internal Cross-Validation								
Model Trained without Outlier Removal	0.894(0.893,0.895)	0.948(0.948,0.950)	0.868(0.868,0.871)	0.902(0.902,0.903)	0.757(0.756,0.760)	0.951(0.951,0.952)	0.809(0.808,0.811)	0.074(0.073,0.074)
Model Trained with Outlier Removal **(17 excluded)**	0.909(0.909,0.910)	0.949(0.949,0.950)	0.876(0.874,0.877)	0.921(0.921,0.922)	0.797(0.796,0.800)	0.955(0.954,0.955)	0.835(0.834,0.836)	0.072(0.071,0.072)
Δ	+0.015	+0.001	+0.008	+0.019	+0.040	+0.004	+0.026	-0.002
Geographical External Validation								
Model Trained without Outlier Removal	0.873(0.871,0.873)	0.926(0.925,0.927)	0.835(0.833,0.837)	0.889(0.887,0.889)	0.756(0.753,0.758)	0.929(0.927,0.929)	0.793(0.791,0.794)	0.092(0.091,0.093)
Model Trained with Outlier Removal **(17 excluded)**	0.864(0.863,0.865)	0.930(0.929,0.931)	0.860(0.858,0.863)	0.865(0.864,0.867)	0.726(0.724,0.729)	0.937(0.936,0.938)	0.787(0.786,0.789)	0.091(0.091,0.092)
Δ	-0.009	+0.004	+0.025	-0.024	-0.030	+0.008	-0.006	-0.001
Temporal External Validation								

Model Trained without outlier removal	0.838(0.834,0.838)	0.927(0.924,0.927)	0.725(0.719,0.727)	0.881(0.878,0.881)	0.698(0.691,0.699)	0.894(0.891,0.895)	0.712(0.703,0.709)	0.105(0.105,0.107)
Model Trained with Outlier Removal **(17 excluded)**	0.838(0.834,0.838)	0.932(0.929,0.932)	0.784(0.777,0.785)	0.858(0.855,0.859)	0.678(0.671,0.679)	0.913(0.910,0.913)	0.727(0.719,0.725)	0.096(0.096,0.097)
Δ	0	+0.005	+0.059	-0.023	-0.020	+0.019	+0.015	-0.009

Note: Seventeen outliers were identified and excluded in the derivation cohort prior to model training. To quantify the effect of this outlier-removal step on model performance, we retrained the model without the outlier-removal step in the derivation cohort. Model performance was then compared between this retrained model (trained without outlier removal) and the original model (trained with outlier removal) across validation cohorts.

Abbreviations: AUROC, area under the receiver operating characteristic curve; PPV, positive predictive value; NPV, negative predictive value; Δ: Model Trained with outlier removal – Model trained with without outlier removal.

Table S4. Youden Index of 15 ML models

Name	Youden Index
GAM	0.220
LR	0.311
GBDT	1.000
KNN	1.000
AdaBoost	0.492
LightGBM	0.263
RotF	0.387
RF	0.366
XGBoost	0.550
GPC	0.419
ExtraTrees	0.402
DT	0.333
NB	0.005

MLP	0.409
SVM	0.152

Abbreviations: GAM, generalized additive model; LR, logistic regression; GBDT, gradient-boosted decision tree; KNN, k-nearest neighbor; LightGBM, light gradient boosting machine; RotF, rotation forest; XGBoost, eXtreme gradient boosting; NB, naive Bayes; AdaBoost, adaptive boosting; MLP, multilayer perceptron; SVM, support vector machine; DT, decision tree; ExtraTrees, extremely randomized trees; GPC, Gaussian process classifier; RF, random forest.

Table S5. Predictive performance of ML models in predicting overall complication

Name	Accuracy	AUROC	TP	TN	FP	FN	Sensitivity	Specificity	PPV	NPV	F1	Brier score
Internal Cross-Validation												
GAM	0.876(0.875,0.876)	0.940(0.939,0.941)	183.000	493.000	77.000	19.000	0.906(0.904,0.906)	0.865(0.864,0.866)	0.704(0.702,0.705)	0.963(0.962,0.963)	0.792(0.790,0.793)	0.076(0.076,0.077)
LR	0.900(0.899,0.901)	0.940(0.939,0.940)	174.000	521.000	49.000	28.000	0.861(0.859,0.862)	0.914(0.913,0.915)	0.780(0.778,0.782)	0.949(0.948,0.949)	0.819(0.817,0.819)	0.079(0.079,0.079)
GBDT	0.877(0.877,0.878)	0.938(0.938,0.939)	180.000	497.000	73.000	22.000	0.891(0.890,0.893)	0.872(0.872,0.873)	0.711(0.710,0.714)	0.958(0.957,0.958)	0.791(0.790,0.793)	0.105(0.104,0.105)
KNN	0.895(0.894,0.895)	0.936(0.935,0.936)	175.000	516.000	54.000	27.000	0.866(0.864,0.867)	0.905(0.905,0.906)	0.764(0.763,0.766)	0.950(0.949,0.950)	0.812(0.810,0.813)	0.076(0.076,0.077)
AdaBoost	0.872(0.871,0.873)	0.939(0.939,0.940)	187.000	486.000	84.000	15.000	0.926(0.925,0.928)	0.853(0.852,0.854)	0.690(0.689,0.692)	0.970(0.970,0.971)	0.791(0.790,0.792)	0.208(0.208,0.208)
LightGBM	0.892(0.892,0.893)	0.941(0.941,0.942)	180.000	509.000	61.000	22.000	0.891(0.891,0.893)	0.893(0.892,0.893)	0.747(0.744,0.747)	0.959(0.959,0.960)	0.813(0.811,0.813)	0.079(0.078,0.079)
RotF	0.858(0.857,0.858)	0.939(0.939,0.940)	189.000	473.000	97.000	13.000	0.936(0.935,0.937)	0.830(0.829,0.831)	0.661(0.658,0.662)	0.973(0.973,0.974)	0.775(0.772,0.775)	0.083(0.082,0.083)
**RF**	**0.909(0.909,0.910)**	**0.949(0.949,0.950)**	**177.000**	**525.000**	**45.000**	**25.000**	**0.876(0.874,0.877)**	**0.921(0.921,0.922)**	**0.797(0.796,0.800)**	**0.955(0.954,0.955)**	**0.835(0.834,0.836)**	**0.072(0.071,0.072)**
XGBoost	0.895(0.894,0.896)	0.938(0.937,0.939)	175.000	516.000	54.000	27.000	0.866(0.866,0.869)	0.905(0.904,0.906)	0.764(0.763,0.766)	0.950(0.950,0.951)	0.812(0.811,0.813)	0.080(0.079,0.080)
GPC	0.878(0.877,0.879)	0.944(0.943,0.944)	182.000	496.000	74.000	20.000	0.901(0.899,0.902)	0.870(0.869,0.871)	0.711(0.708,0.712)	0.961(0.961,0.962)	0.795(0.792,0.795)	0.083(0.083,0.084)
ExtraTrees	0.907(0.907,0.908)	0.951(0.950,0.951)	174.000	526.000	44.000	28.000	0.861(0.861,0.864)	0.923(0.923,0.924)	0.798(0.798,0.801)	0.949(0.949,0.950)	0.829(0.828,0.830)	0.073(0.073,0.073)
DT	0.861(0.861,0.863)	0.873(0.872,0.874)	168.000	497.000	73.000	34.000	0.832(0.830,0.834)	0.872(0.872,0.873)	0.697(0.697,0.701)	0.936(0.935,0.937)	0.758(0.758,0.761)	0.102(0.101,0.102)
NB	0.858(0.857,0.859)	0.904(0.903,0.905)	181.000	481.000	89.000	21.000	0.896(0.895,0.898)	0.844(0.843,0.845)	0.670(0.670,0.674)	0.958(0.958,0.959)	0.767(0.767,0.769)	0.136(0.136,0.137)
MLP	0.878(0.878,0.879)	0.917(0.916,0.918)	173.000	505.000	65.000	29.000	0.856(0.856,0.859)	0.886(0.885,0.887)	0.727(0.725,0.729)	0.946(0.945,0.947)	0.786(0.785,0.788)	0.093(0.092,0.093)
SVM	0.881(0.880,0.882)	0.941(0.940,0.942)	187.000	493.000	77.000	15.000	0.926(0.923,0.926)	0.865(0.865,0.866)	0.708(0.707,0.710)	0.970(0.970,0.971)	0.803(0.801,0.803)	0.076(0.075,0.076)

Geographical External Validation												
GAM	0.851(0.849,0.851)	0.927(0.926,0.928)	98.000	233.000	42.000	16.000	0.860(0.856,0.860)	0.847(0.845,0.848)	0.700(0.696,0.701)	0.936(0.934,0.936)	0.772(0.768,0.771)	0.091(0.091,0.092)
LR	0.853(0.853,0.855)	0.921(0.919,0.921)	97.000	235.000	40.000	17.000	0.851(0.848,0.852)	0.855(0.854,0.856)	0.708(0.707,0.712)	0.933(0.931,0.933)	0.773(0.771,0.775)	0.098(0.098,0.099)
GBDT	0.807(0.807,0.809)	0.911(0.909,0.911)	43.000	271.000	4.000	71.000	0.377(0.374,0.380)	0.985(0.985,0.986)	0.915(0.914,0.919)	0.792(0.792,0.794)	0.534(0.530,0.536)	0.150(0.149,0.151)
KNN	0.776(0.776,0.779)	0.922(0.921,0.923)	27.000	275.000	0.000	87.000	0.237(0.234,0.239)	1.000(1.000,1.000)	1.000(1.000,1.000)	0.760(0.760,0.763)	0.383(0.378,0.384)	0.092(0.091,0.092)
AdaBoost	0.874(0.873,0.875)	0.929(0.929,0.930)	94.000	246.000	29.000	20.000	0.825(0.824,0.828)	0.895(0.893,0.896)	0.764(0.762,0.766)	0.925(0.924,0.926)	0.793(0.791,0.795)	0.208(0.208,0.208)
LightGBM	0.835(0.833,0.836)	0.928(0.927,0.928)	99.000	226.000	49.000	15.000	0.868(0.866,0.870)	0.822(0.819,0.822)	0.669(0.665,0.670)	0.938(0.937,0.938)	0.756(0.752,0.756)	0.096(0.096,0.097)
RotF	0.843(0.843,0.845)	0.921(0.920,0.921)	92.000	236.000	39.000	22.000	0.807(0.802,0.807)	0.858(0.859,0.861)	0.702(0.701,0.706)	0.915(0.913,0.915)	0.751(0.748,0.752)	0.099(0.098,0.100)
**RF**	**0.864(0.863,0.865)**	**0.930(0.929,0.931)**	**98.000**	**238.000**	**37.000**	**16.000**	**0.860(0.858,0.863)**	**0.865(0.864,0.867)**	**0.726(0.724,0.729)**	**0.937(0.936,0.938)**	**0.787(0.786,0.789)**	**0.091(0.091,0.092)**
XGBoost	0.877(0.876,0.878)	0.926(0.926,0.927)	86.000	255.000	20.000	28.000	0.754(0.753,0.758)	0.927(0.926,0.928)	0.811(0.809,0.813)	0.901(0.901,0.903)	0.782(0.780,0.783)	0.102(0.101,0.103)
GPC	0.866(0.865,0.867)	0.920(0.918,0.921)	95.000	242.000	33.000	19.000	0.833(0.831,0.836)	0.880(0.878,0.880)	0.742(0.739,0.744)	0.927(0.926,0.928)	0.785(0.782,0.786)	0.102(0.101,0.102)
ExtraTrees	0.853(0.852,0.855)	0.923(0.922,0.924)	94.000	238.000	37.000	20.000	0.825(0.824,0.828)	0.865(0.864,0.866)	0.718(0.715,0.720)	0.922(0.922,0.924)	0.767(0.765,0.769)	0.098(0.098,0.099)
DT	0.843(0.843,0.845)	0.862(0.861,0.864)	89.000	239.000	36.000	25.000	0.781(0.781,0.785)	0.869(0.868,0.871)	0.712(0.710,0.715)	0.905(0.905,0.907)	0.745(0.743,0.747)	0.117(0.115,0.117)
NB	0.739(0.739,0.741)	0.771(0.770,0.772)	222.000	340.000	39.000	159.000	0.583(0.582,0.585)	0.897(0.896,0.898)	0.851(0.850,0.853)	0.681(0.680,0.683)	0.692(0.691,0.693)	0.192(0.192,0.193)
MLP	0.838(0.838,0.839)	0.851(0.850,0.852)	353.000	284.000	95.000	28.000	0.927(0.925,0.927)	0.749(0.749,0.751)	0.788(0.787,0.790)	0.910(0.909,0.911)	0.852(0.851,0.852)	0.225(0.225,0.225)
SVM	0.783(0.782,0.784)	0.858(0.857,0.859)	292.000	303.000	76.000	89.000	0.766(0.764,0.767)	0.799(0.799,0.801)	0.793(0.793,0.795)	0.773(0.771,0.774)	0.780(0.778,0.781)	0.155(0.154,0.155)

Abbreviations: AUROC, area under the receiver operating characteristic curve; TP, true positive; TN, true negative; FP, false positive; FN, false negative; PPV, positive predictive value; NPV, negative predictive value; GAM, generalized additive model; LR, logistic regression; GBDT, gradient-boosted decision tree; KNN, k-nearest neighbor; LightGBM, light gradient boosting machine; RotF, rotation forest; XGBoost, eXtreme gradient boosting; NB, naive Bayes; AdaBoost, adaptive boosting; MLP, multilayer perceptron; SVM, support vector machine; DT, decision tree; ExtraTrees, extremely randomized trees; GPC, Gaussian process classifier; RF, random forest.

Table S6. Predictive performance of ML models in predicting specific complications

**Name**	**Accuracy**	**AUROC**	**TP**	**TN**	**FP**	**FN**	**Sensitivity**	**Specificity**	**PPV**	**NPV**	**F1**	**Brier score**
Infection												

5-Fold Cross-Validation	0.908(0.908,0.909)	0.940(0.940,0.940)	745	622	128	10	0.987(0.986,0.987)	0.829(0.829,0.830)	0.853(0.853,0.854)	0.984(0.984,0.984)	0.915(0.915,0.916)	0.082(0.082,0.083)
Geographical External Validation	0.808(0.807,0.808)	0.872(0.870,0.872)	381	233	146	0	1.000(1.000,1.000)	0.615(0.613,0.616)	0.723(0.721,0.724)	1.000(1.000,1.000)	0.839(0.838,0.840)	0.152(0.151,0.152)
Fluid Collections												
5-Fold Cross-Validation	0.895(0.895,0.896)	0.939(0.939,0.940)	617	580	82	58	0.914(0.912,0.914)	0.876(0.877,0.878)	0.883(0.883,0.884)	0.909(0.908,0.909)	0.898(0.898,0.899)	0.089(0.088,0.089)
Geographical External Validation	0.817(0.816,0.818)	0.884(0.883,0.884)	265	235	72	40	0.869(0.868,0.870)	0.765(0.764,0.767)	0.786(0.785,0.788)	0.855(0.853,0.856)	0.826(0.824,0.826)	0.141(0.141,0.142)
Pneumocephalus												
5-Fold Cross-Validation	0.883(0.882,0.883)	0.927(0.926,0.927)	627	554	108	48	0.929(0.928,0.929)	0.837(0.834,0.836)	0.853(0.851,0.853)	0.920(0.919,0.921)	0.889(0.888,0.889)	0.099(0.099,0.100)
Geographical External Validation	0.791(0.790,0.792)	0.859(0.858,0.860)	230	254	53	75	0.754(0.752,0.755)	0.827(0.827,0.830)	0.813(0.812,0.815)	0.772(0.771,0.774)	0.782(0.781,0.783)	0.165(0.165,0.166)
Intracranial Hemorrhage												
5-Fold Cross-Validation	0.959(0.959,0.959)	0.989(0.989,0.989)	718	704	35	26	0.965(0.965,0.966)	0.953(0.953,0.954)	0.954(0.954,0.954)	0.964(0.964,0.965)	0.959(0.959,0.960)	0.046(0.046,0.046)
Geographical External Validation	0.848(0.847,0.848)	0.919(0.919,0.920)	304	324	46	67	0.819(0.819,0.821)	0.876(0.875,0.877)	0.869(0.867,0.870)	0.829(0.828,0.830)	0.843(0.842,0.844)	0.121(0.121,0.121)
Hydrocephalus												
5-Fold Cross-Validation	0.941(0.940,0.941)	0.970(0.969,0.970)	730	665	74	14	0.981(0.981,0.982)	0.900(0.898,0.900)	0.908(0.907,0.908)	0.979(0.979,0.980)	0.943(0.942,0.943)	0.051(0.051,0.051)
Geographical External Validation	0.838(0.837,0.839)	0.904(0.903,0.905)	297	324	46	74	0.801(0.799,0.801)	0.876(0.875,0.877)	0.866(0.865,0.867)	0.814(0.812,0.815)	0.832(0.830,0.832)	0.152(0.151,0.153)
Seizures												
5-Fold Cross-Validation	0.857(0.857,0.858)	0.913(0.912,0.913)	652	633	115	99	0.868(0.868,0.869)	0.846(0.845,0.846)	0.850(0.849,0.851)	0.865(0.864,0.865)	0.859(0.859,0.860)	0.118(0.118,0.118)
Geographical External Validation	0.897(0.897,0.898)	0.939(0.939,0.940)	354	318	57	20	0.947(0.946,0.947)	0.848(0.847,0.850)	0.861(0.861,0.863)	0.941(0.940,0.941)	0.902(0.902,0.903)	0.100(0.100,0.100)
Reoperations												
5-Fold Cross-Validation	0.984(0.984,0.984)	0.997(0.997,0.997)	748	741	18	6	0.992(0.992,0.992)	0.976(0.976,0.976)	0.977(0.976,0.977)	0.992(0.992,0.992)	0.984(0.984,0.984)	0.021(0.021,0.022)
Geographical External Validation	0.872(0.871,0.873)	0.928(0.927,0.929)	354	311	70	28	0.927(0.926,0.927)	0.816(0.816,0.818)	0.835(0.834,0.837)	0.917(0.916,0.918)	0.878(0.878,0.879)	0.115(0.114,0.115)

Abbreviations: AUROC, area under the receiver operating characteristic curve; TP, true positive; TN, true negative; FP, false positive; FN, false negative; PPV, positive predictive value; NPV, negative predictive value.

Table S7. Predictive Performance of Machine Learning Models for Specific Complications in the Temporal External Validation Cohort

Name	Accuracy	AUROC	TP	TN	FP	FN	Sensitivity	Specificity	AUPRC
Infection	0.989(0.989,0.990)	0.901(0.895,0.907)	4	179	1	1	0.800(0.788,0.812)	0.994(0.994,0.995)	0.803(0.801,0.815)
Fluid Collections	0.897(0.895,0.898)	0.935(0.933,0.936)	25	141	4	15	0.625(0.615,0.625)	0.972(0.972,0.973)	0.784(0.782,0.787)
Pneumocephalus	0.957(0.956,0.958)	0.851(0.845,0.856)	8	169	6	2	0.800(0.790,0.807)	0.966(0.965,0.967)	0.691(0.686,0.695)
Intracranial Hemorrhage	0.784(0.781,0.785)	0.952(0.949,0.953)	3	142	40	0	1.000(1.000,1.000)	0.780(0.777,0.781)	0.536(0.536,0.538)
Hydrocephalus	0.876(0.874,0.877)	0.977(0.976,0.978)	4	158	23	0	1.000(1.000,1.000)	0.873(0.871,0.874)	0.574(0.574,0.577)
Seizures	0.870(0.868,0.872)	0.986(0.985,0.986)	6	155	24	0	1.000(1.000,1.000)	0.866(0.864,0.867)	0.600(0.598,0.601)
Reoperations	0.989(0.988,0.990)		0	183	2	0		0.989(0.989,0.990)	

Abbreviations: AUROC, area under the receiver operating characteristic curve; TP, true positive; TN, true negative; FP, false positive; FN, false negative. AUPRC, area under the precision–recall curve.

Table S8. Estimated Average Treatment Effects (ATE) of Surgical Variables with Robustness Checks

Treatment	ATE	Lower_CI	Upper_CI	ATE with Random Perturbation	*p* Value (Robustness Checks)
N-P drainage	-0.241	-0.35	-0.132	-0.264	1
Titanium	-0.191	-0.341	-0.041	-0.173	1

Statistical comparison between ATE and Perturbation ATE was performed using the Mann-Whitney U test, with *p* values < 0.05 indicating statistical significance was considered statistically significant. Abbreviations: ATE, average treatment effect; N-P drainage, postoperative placement of subcutaneous negative-pressure drainage tubes.

Table S9. Estimated Conditional Average Treatment Effect (CATE) of Surgical Variables with Robustness Checks

Treatment	Age	Sex	CATE (Mean)	Lower_CI	Upper_CI	*p* Value (Robustness Checks)

N-P drainage	Over40	Female	-0.279	-0.310	-0.249	0.200
N-P drainage	Over40	Male	-0.047	-0.063	-0.030	0.648
N-P drainage	Under40	Female	-0.213	-0.258	-0.173	0.412
N-P drainage	Under40	Male	-0.164	-0.191	-0.136	0.296
Titanium	Over40	Female	-0.102	-0.123	-0.081	0.318
Titanium	Over40	Male	-0.128	-0.138	-0.119	0.212
Titanium	Under40	Female	-0.099	-0.138	-0.060	0.181
Titanium	Under40	Male	0.009	-0.003	0.021	0.396

Statistical comparison between CATE and Perturbation CATE was performed using the Mann-Whitney U test, with p values < 0.05 indicating statistical significance was considered statistically significant. Abbreviations: CATE, conditional average treatment effect; N-P drainage, postoperative placement of subcutaneous negative-pressure drainage tubes.

Supplementary Figures

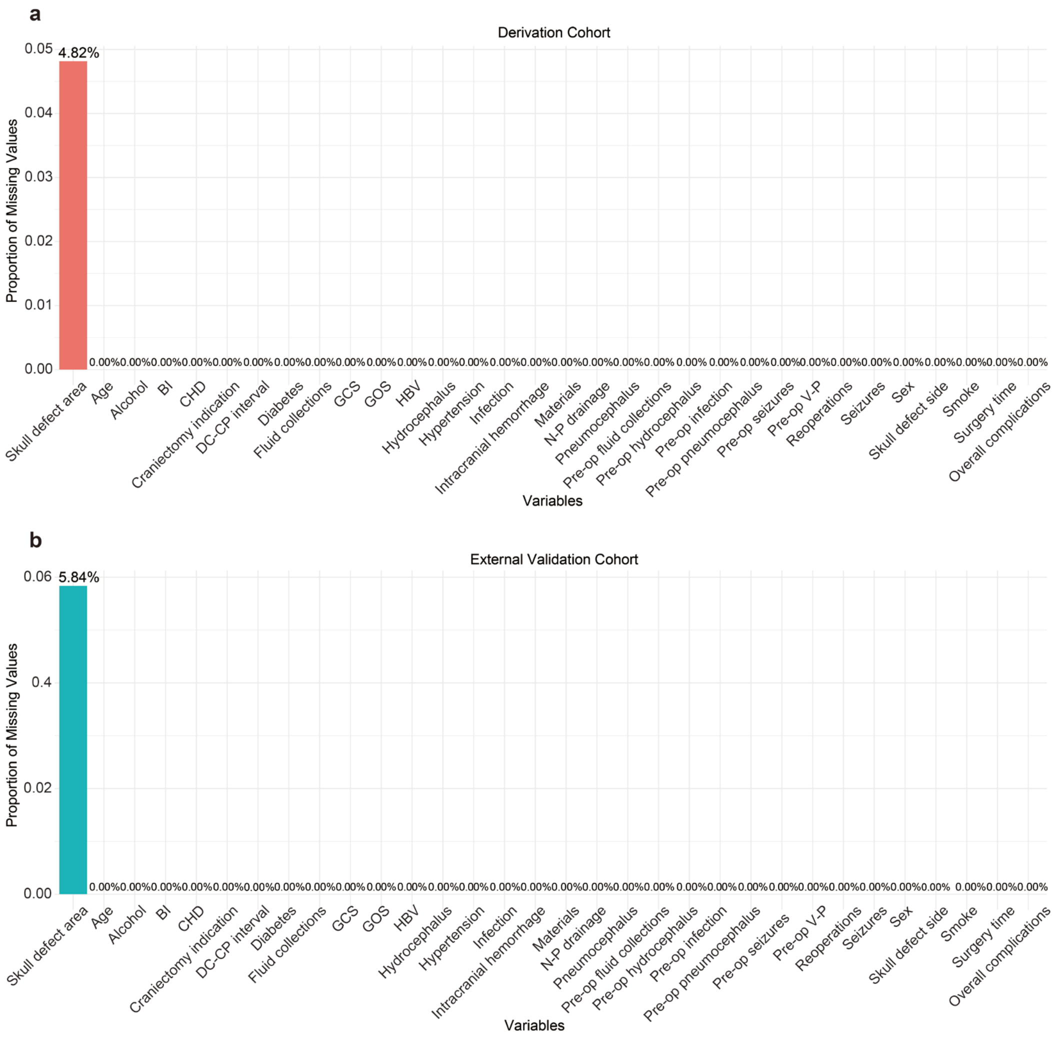

Figure S1. Proportion of missing values across variables in the derivation cohort and external validation cohort.

(A) Proportion of missing values visualized in the derivation cohort. **(B)** Proportion of missing values visualized in the external validation cohort.

The missing rate below 5% is defined as minor missingness, while the missing rate between 5% and 10% is classified as moderate missingness. **Abbreviations:** DC-CP interval, the time interval (in months) between decompressive craniectomy (DC) and cranioplasty (CP); GOS, Glasgow Outcome Scale; GCS, Glasgow Coma Scale; BI, Barthel Index; HBV, hepatitis B virus infection; CHD, coronary heart disease; N-P drainage, postoperative placement of subcutaneous negative-pressure drainage tubes; Pre-op V-P, preoperative ventriculoperitoneal shunt status; Pre-op, preoperative.

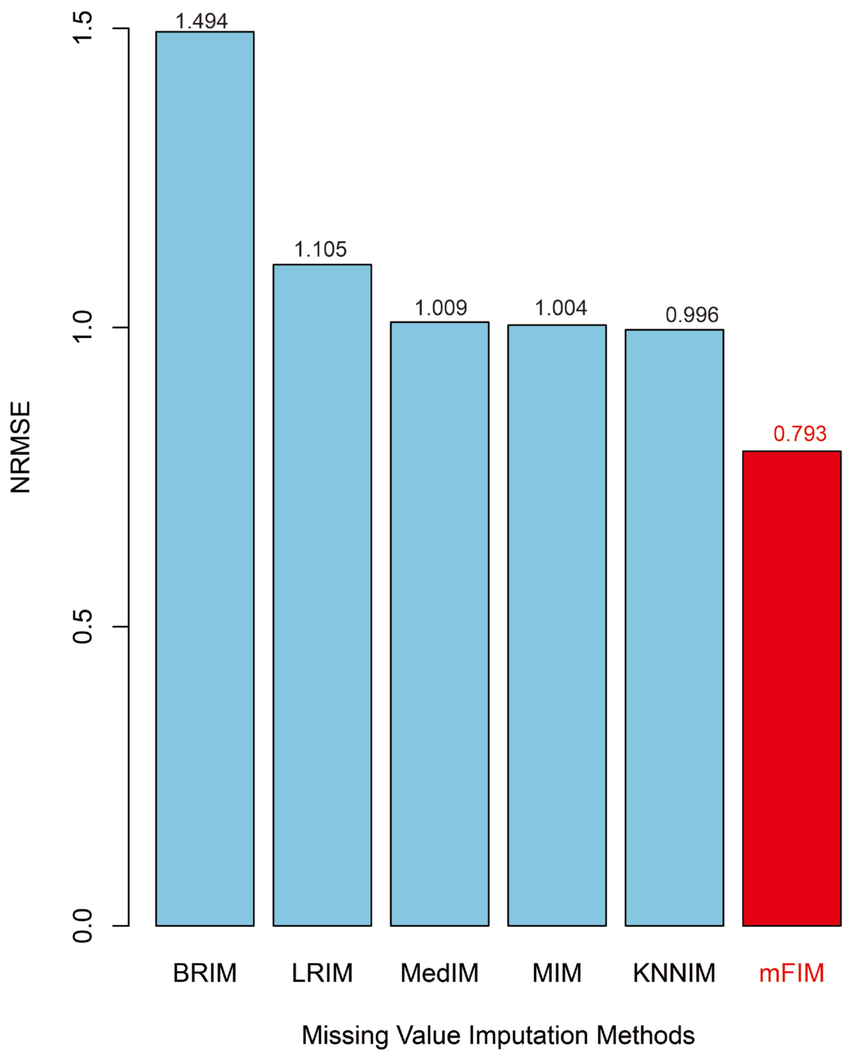

Figure S2. NRMSE values of each imputation method

the normalized root mean square error (NRMSE) was calculated using the following formula:

$$NRMSE = \frac{\sqrt{\frac{1}{n}\sum_{i=1}^{n}(\hat{x}_i - x_i)^2}}{\sigma}$$

where $\hat{x}_i$ represents the imputed value; x_i represents the true observed value; n represents the number of imputed values; σ is the standard deviation of the true observed values. The lowest weighted average NRMSE was selected as the optimal imputation approach for handling missing data in this study. **Abbreviations:** BRIM, Bayesian regression imputation method; LRIM, linear regression imputation method; MedIM, median imputation method; MIM, mean imputation method; KNNIM, k-nearest neighbors imputation method; mFIM, missForest imputation method.

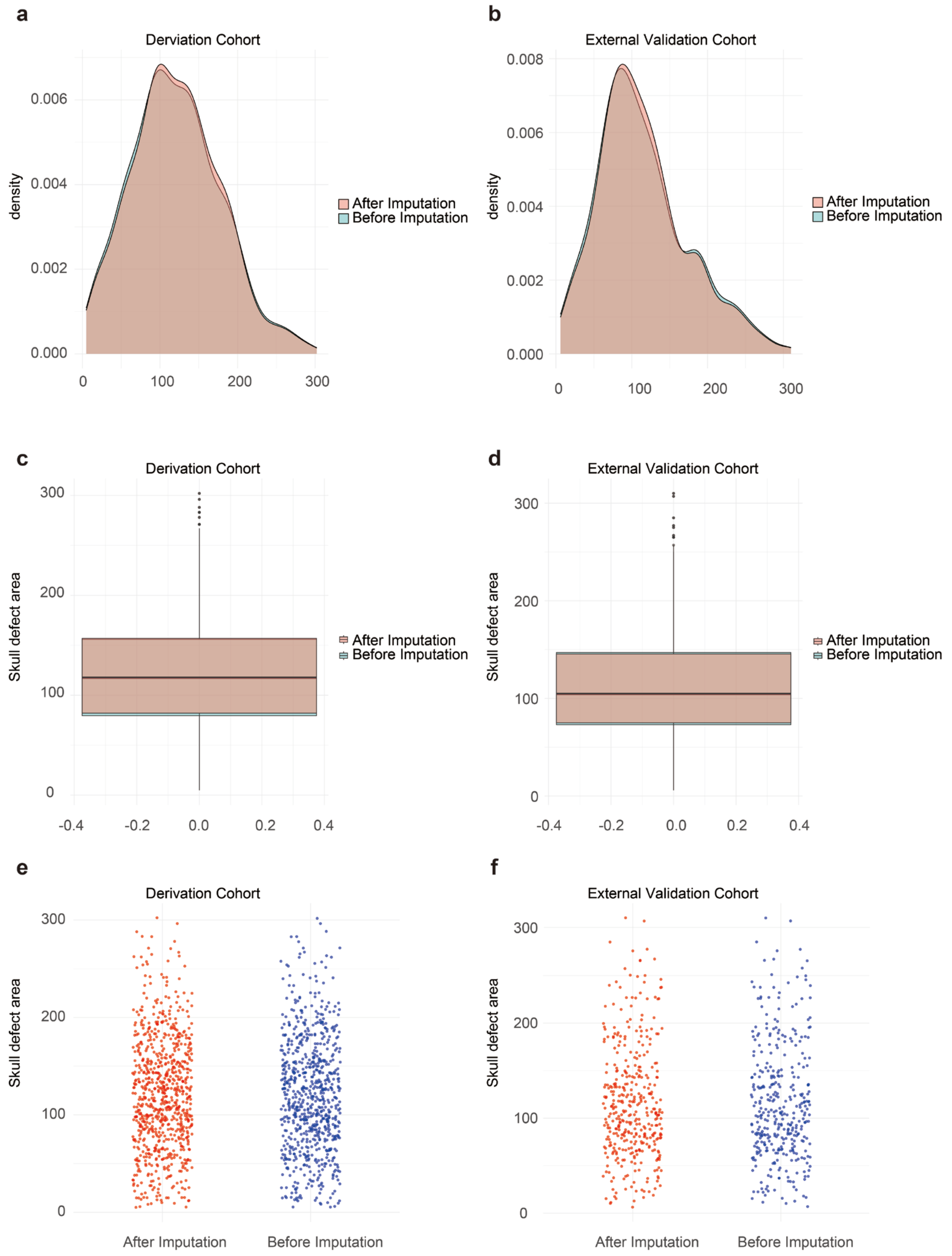

Figure S3. Sensitivity analysis of imputation results

(a) Density plot comparing data before and after imputation in the derivation cohort. **(b)** Density plot comparing data before and after imputation in the external validation cohort. **(c)** Box plot comparing data before and after imputation in the derivation cohort. **(d)** Box plot comparing data before and after imputation in the external validation cohort. **(e)** Scatter plot comparing data before and after imputation in the derivation cohort. **(f)** Scatter plot comparing data before and after imputation in the external validation cohort.

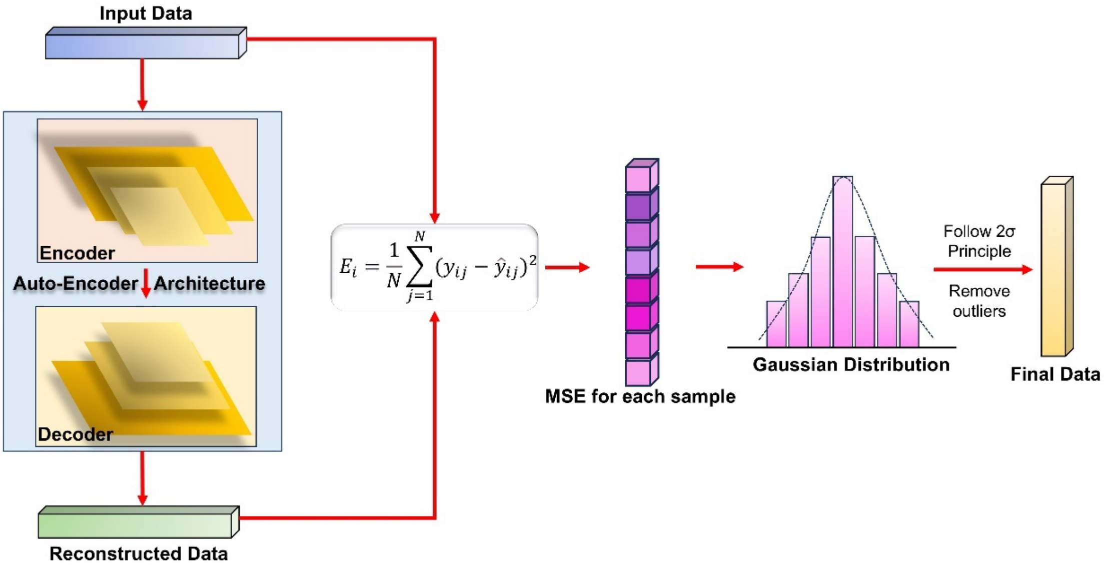

Figure S4. Workflow of the designed autoencoder for outlier detection

E_i represents the reconstruction error for the i-th sample. It quantifies the average discrepancy between the input data and its reconstructed output, reflecting the autoencoder's ability to represent this sample; N represents the total number of features for each sample; $(y_{ij} - \hat{y}_{ij})^2$ represents the squared difference between the observed value y_{ij} and the corresponding reconstructed value $\hat{y}_{ij}$.

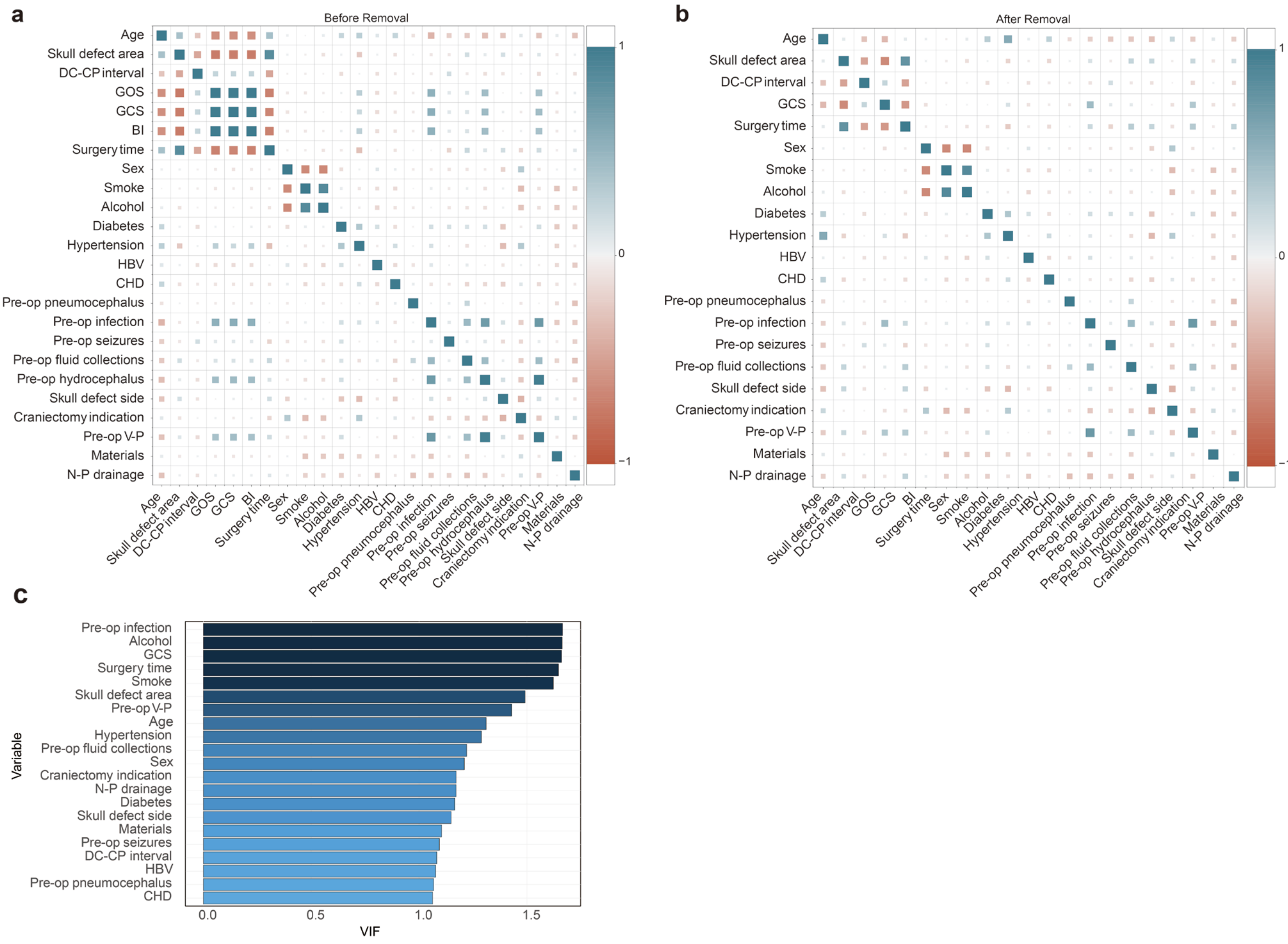

Figure S5. Correlation filtering of highly correlated variables

(a) Heatmap of correlation analyses among variables before correlation filtering. **(b)** Heatmap of correlation analyses among variables after correlation filtering. **(c)** Variance inflation factor (VIF) for the remaining variables.

Abbreviations: DC-CP interval, the time interval (in months) between decompressive craniectomy (DC) and cranioplasty (CP); GOS, Glasgow Outcome Scale; GCS, Glasgow Coma Scale; BI, Barthel Index; HBV, hepatitis B virus infection; CHD, coronary heart disease; N-P drainage, postoperative placement of negative-pressure drainage tubes; craniectomy indication; Pre-op V-P, preoperative ventriculoperitoneal shunt status; Pre-op, preoperative.

The filtering process involves the following steps:

(1) Calculate the correlation coefficient matrix of the variables.
(2) Identify the pair of variables with the highest absolute correlation coefficient (denoted as variables A and B).
(3) Calculate the correlation coefficients of A and B with all other predictor variables separately.
(4) If the average correlation coefficient of A is greater, remove variable A; otherwise, remove variable B.
(5) Repeat steps 2-4 until the absolute value of all correlation coefficients falls below 0.6.

The correlation coefficients are calculated as follows:

(1) For continuous and ordered categorical variables, use Spearman's Rank Correlation Coefficient.

$$\rho = 1 - \frac{6\sum d_i^2}{n(n^2 - 1)}$$

where ρ is Spearman's rank correlation coefficient; d_i is the difference between the ranks of each pair of observation; n is the number of observations.

(2) For unordered categorical variables, apply Theil's U,

$$H(X) = -\sum_{i=1}^{n} p(x_i)\log\big(p(x_i)\big)$$

$$H(X|Y) = -\sum_{y \in Y}\sum_{x \in X} p(x,y)\log\,(p(x|y))$$

$$U = \frac{H(X) - H(X|Y)}{H(X)}$$

where $H(X)$ is the entropy of variable X; $p(x_i)$ is the probability of category x_i; $p(x,y)$ is the joint probability of category x and category y occurring together; n is the total number of categories; U is the Theil's U; $H(X|Y)$ is the conditional entropy of X given Y.

(3) For correlations involving unordered categorical variables and continuous or ordered categorical variables, employ Correlation Ratios.

$$\eta^2 = \frac{SS(Between\ groups)}{SS(Total)}$$

where $SS(Between\ groups)$ is the sum of squares for the effect of the independent variable and $SS(Total)$ is the total sum of squares.

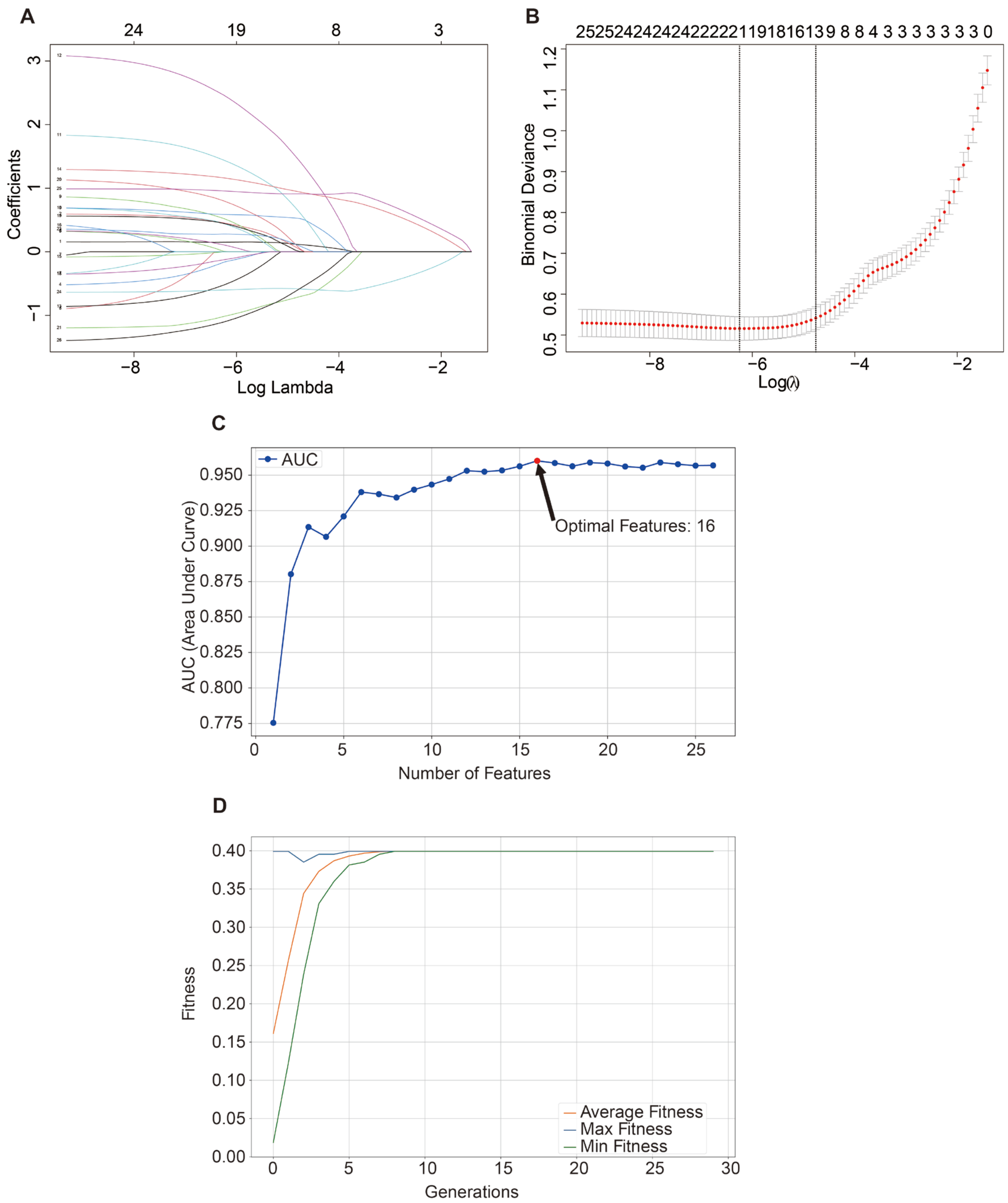

Figure S6. Variable selection using Least Absolute Shrinkage and Selection Operator (LASSO), Recursive Feature Elimination (RFE), and genetic algorithm (GA)

(a) Cross-validation error plot for LASSO. **(b)** Coefficient path plot for LASSO. The optimal λ (λ_{min}) determined via 10-fold cross-validation. **(c)** Illustration of Recursive Feature Elimination (RFE) for variable selection. A Random Forest model was trained to evaluate feature importance, and the optimal subset was selected via 10-fold cross-validation. **(d)** Fitness convergence plot for the genetic algorithm (GA). The fitness function was defined as

$$\text{Fitness} = \sum_{j \in S} IG_j - \alpha \times |S|$$

where S is the set of selected features; IG_j is the information gain of feature j; $|S|$ is the number of selected features, and α is the penalty coefficient to control model complexity.

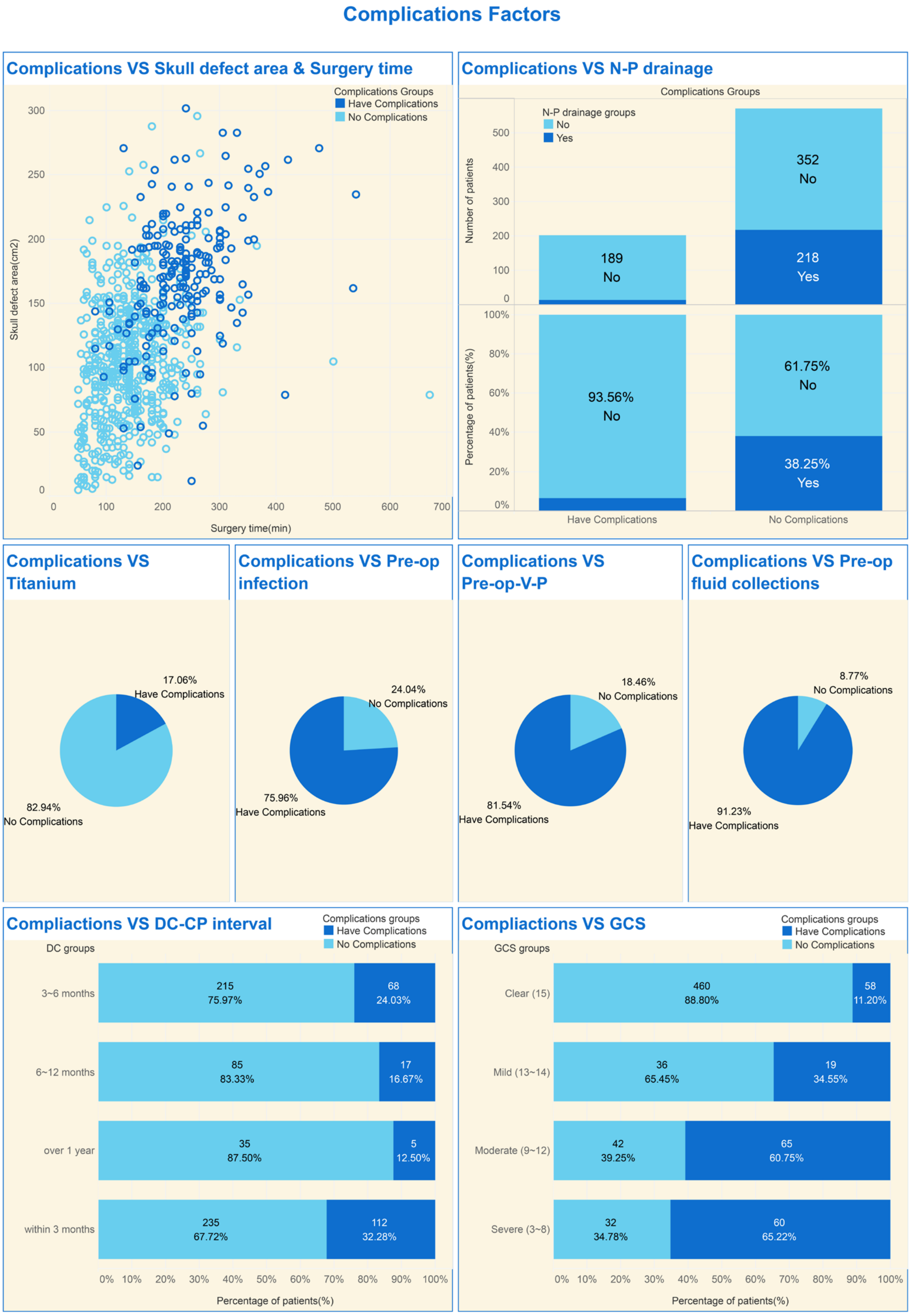

Figure S7. Distribution of the Nine Selected Predictors Stratified by Postoperative Complication Status

Abbreviations: DC-CP interval, the time interval (in months) between decompressive craniectomy (DC) and cranioplasty (CP); GCS, Glasgow Coma Scale; N-P drainage, postoperative placement of subcutaneous negative-pressure drainage tubes; craniectomy indication; Pre-op V-P, preoperative ventriculoperitoneal shunt status; Pre-op, preoperative.

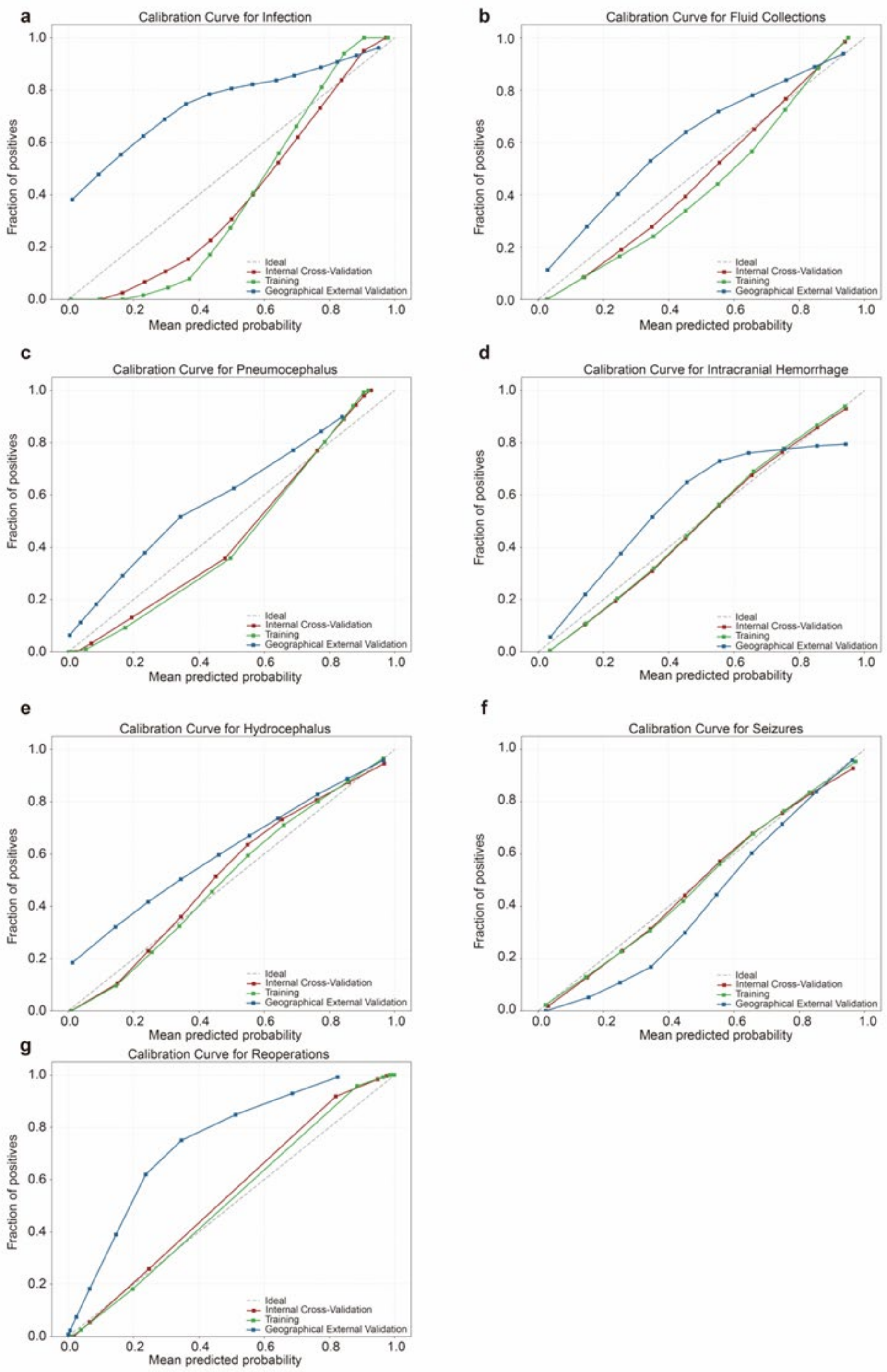

Figure S8. Calibration curves of the best-performing machine learning models for predicting each complication.

(a) Infection. (**b)** Fluid collections. **(c)** Pneumocephalus. **(d)** Intracranial hemorrhage. **(e)** Hydrocephalus. **(f)** Seizures. **(g)** Reoperations. **Note:** The calibration curves were smoothed using LOESS (locally weighted scatterplot smoothing).

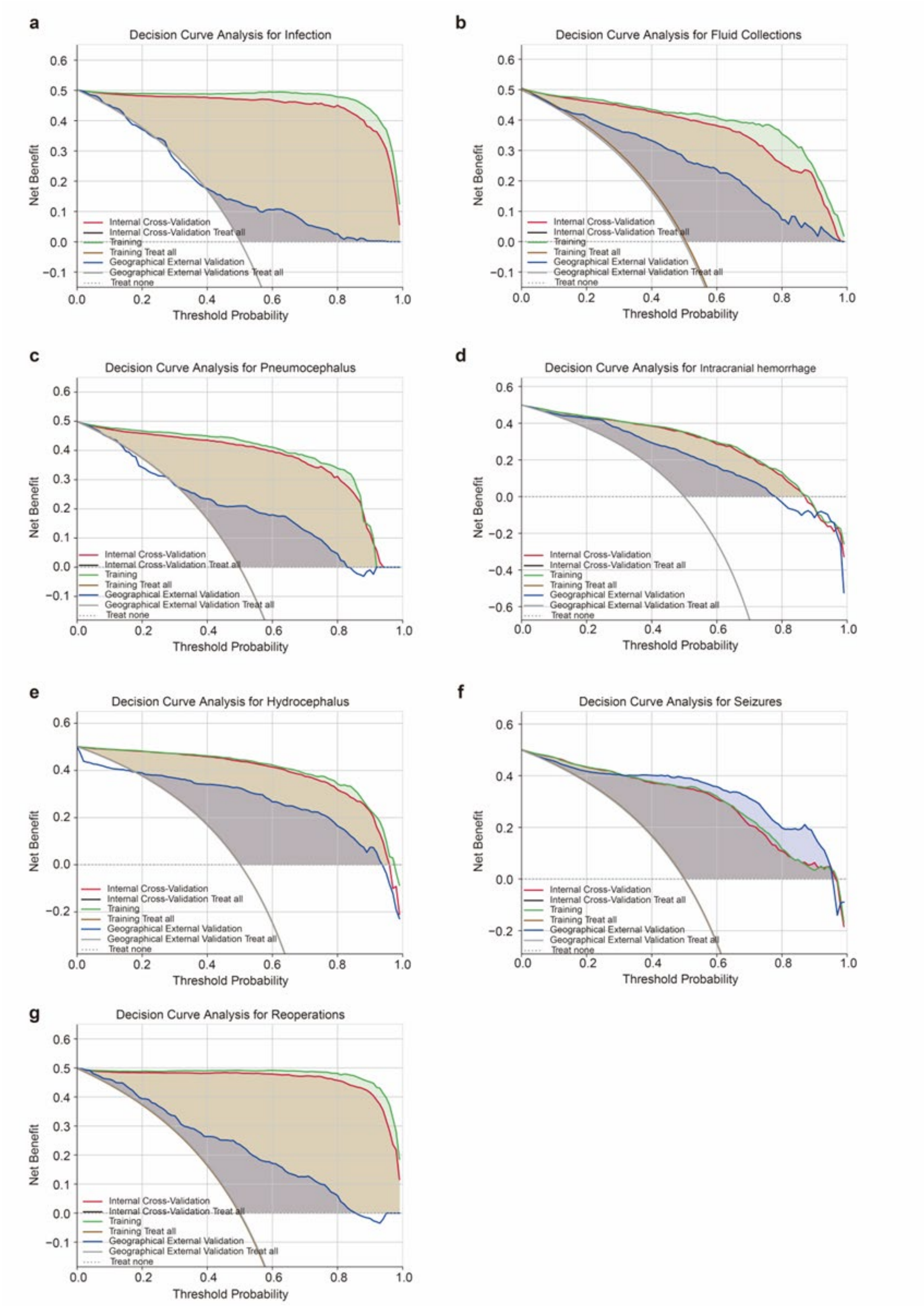

Figure S9. Decision curves of the best-performing machine learning models for predicting each complication.

(a) Infection. (**b)** Fluid collections. **(c)** Pneumocephalus. **(d)** Intracranial hemorrhage. **(e)** Hydrocephalus. **(f)** Seizures. **(g)** Reoperations.

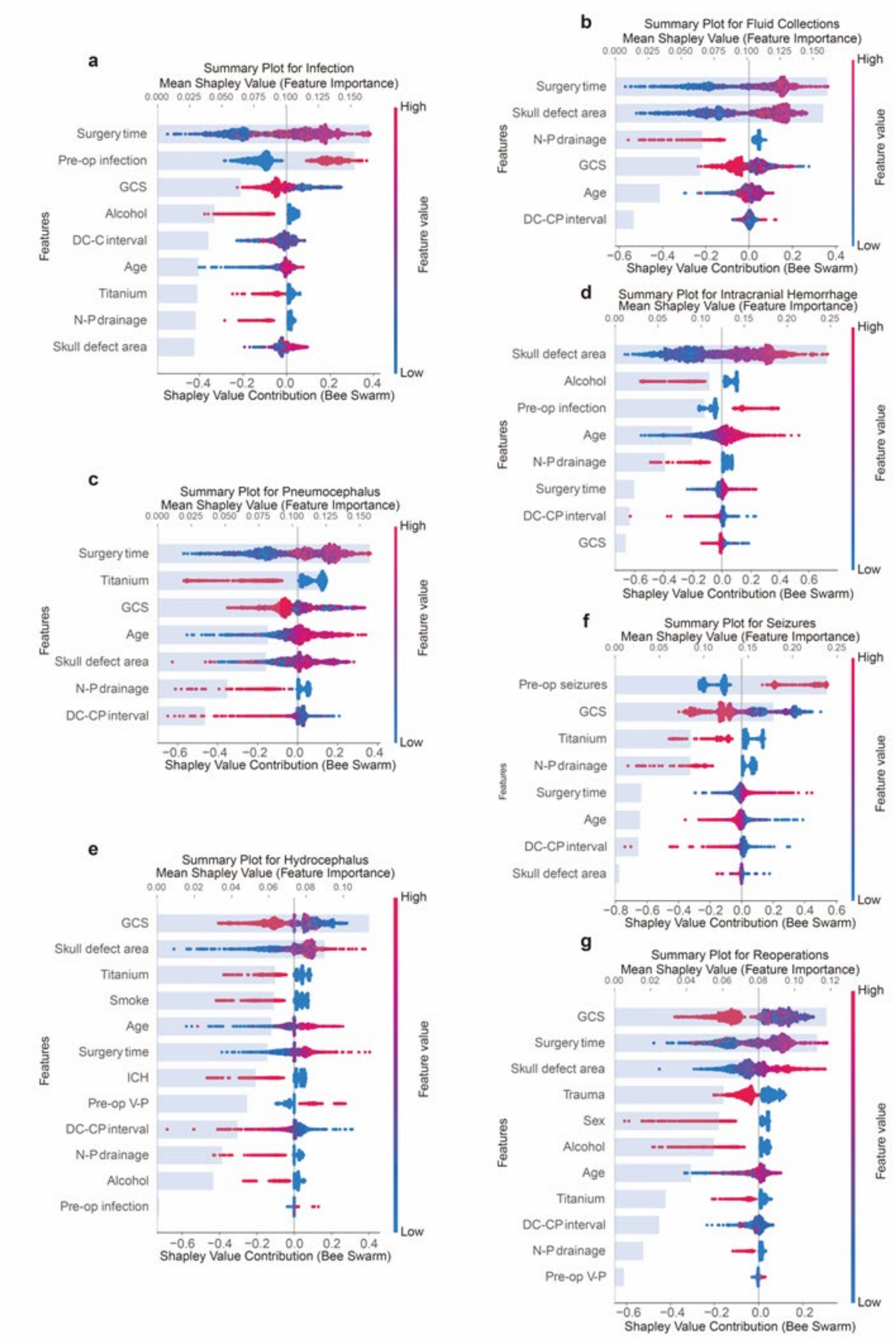

Figure S10. SHAP summary plots of the best-performing machine learning models for predicting each complication.

(a) Infection. **(b)** Fluid collections. **(c)** Pneumocephalus. **(d)** Intracranial hemorrhage. **(e)** Hydrocephalus. **(f)** Seizures. **(g)** Reoperations.

Abbreviations: DC-CP interval, the time interval (in months) between decompressive craniectomy (DC) and cranioplasty (CP); GCS, Glasgow Coma Scale; N-P drainage, postoperative placement of subcutaneous negative-pressure drainage tubes; Pre-op V-P, preoperative ventriculoperitoneal shunt status; Pre-op, preoperative; ICH, intracranial hemorrhage.

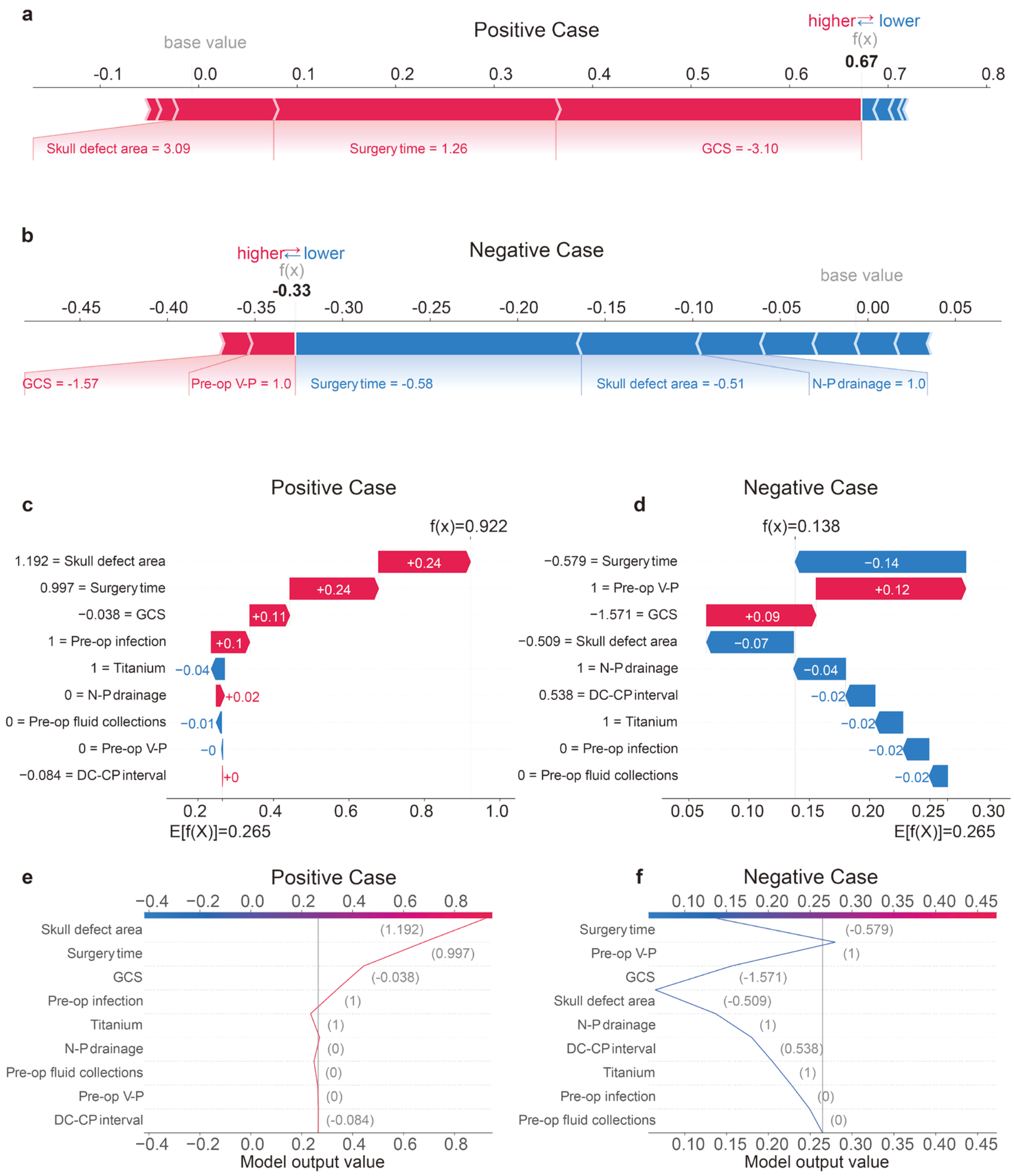

Figure S11. Local SHAP explanations for representative samples

(a–b) SHAP force plots showing the feature contributions to model prediction in a representative positive case **(a)** and a negative case **(b)**. **(c–d)** SHAP waterfall plots illustrating how each feature contributes to the model's prediction in a representative positive case **(c)** and a negative case **(d)**. **(e–f)** SHAP decision plots illustrating the cumulative effect of each variable on the final prediction in a representative positive case **(e)** and a negative case **(f).**

a

b

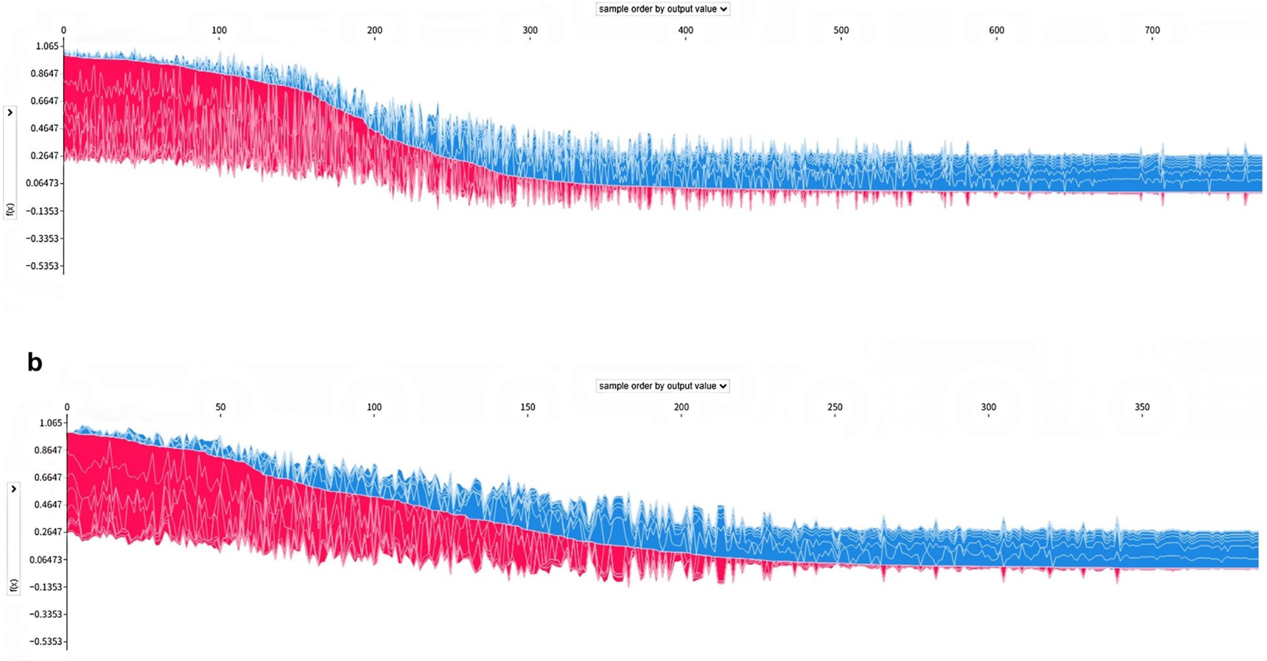

Figure S12. Local SHAP explanations by the SHAP method.

(a) Force plot for the derivation cohort. **(b)** Force plot for the geographical external validation cohort. Each patient was represented by the x-axis, while the features' contributions were represented by the y-axis: a larger red segment for each individual patient indicated a higher predicted probability of complication after cranioplasty.

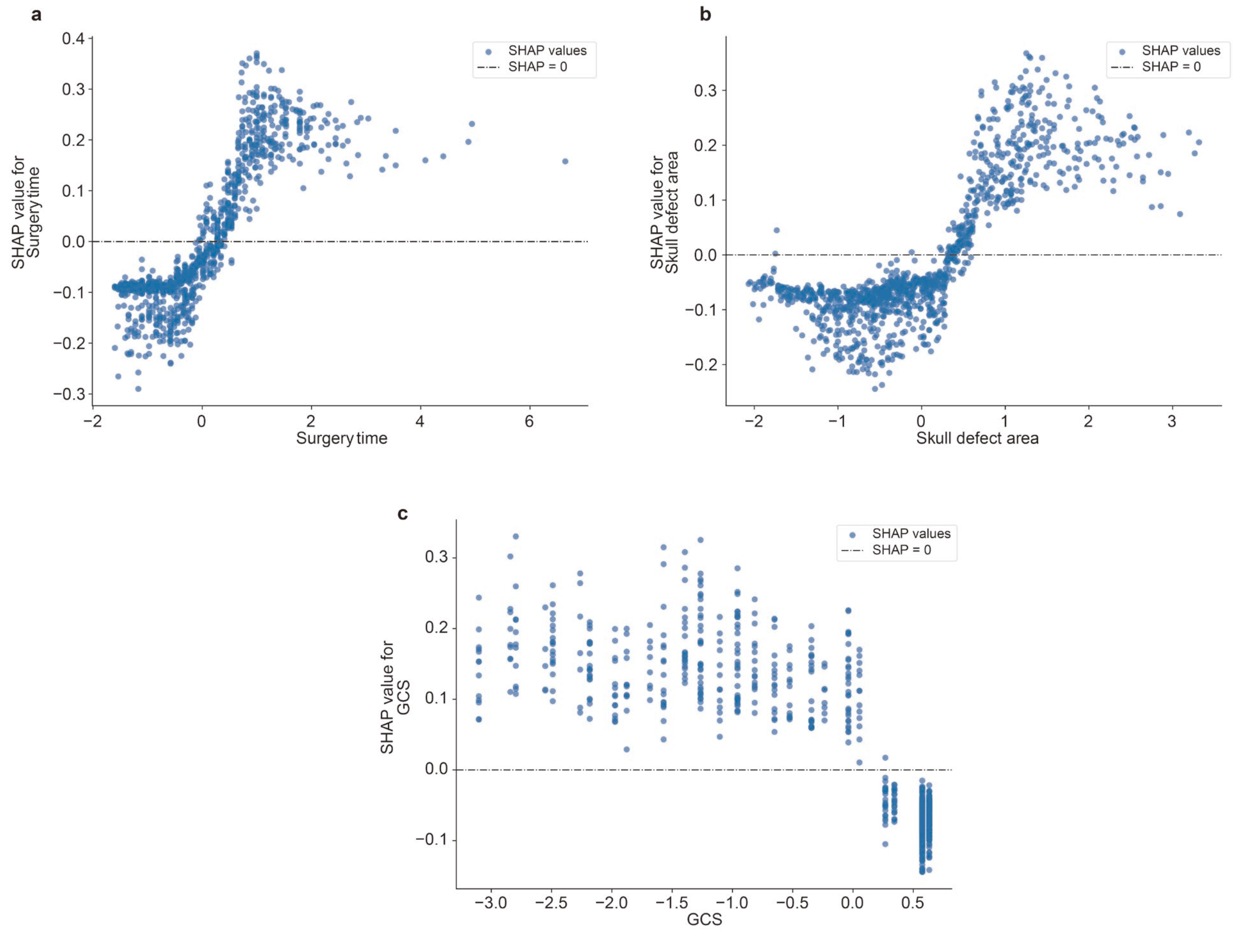

Figure S13. SHAP dependence plots of key predictive features

(a) Surgery time. **(b)** Skull defect area. **(c)** Glasgow Coma Scale (GCS)

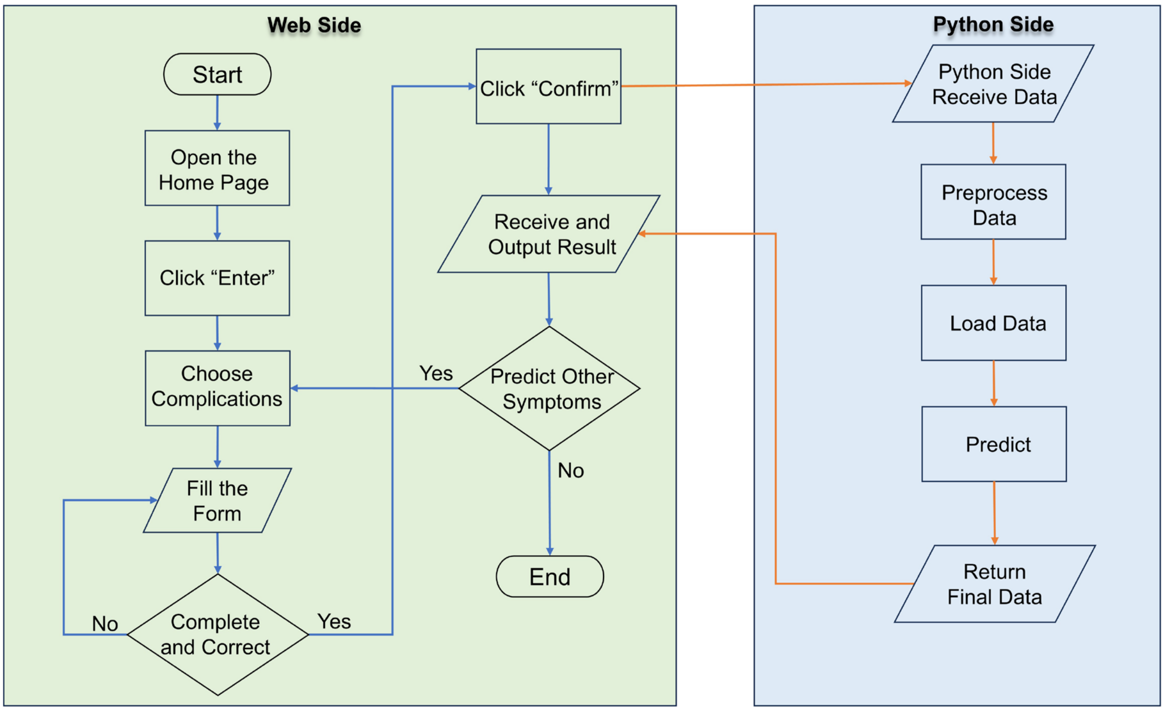

Figure S14. Flow chart of web deployment.

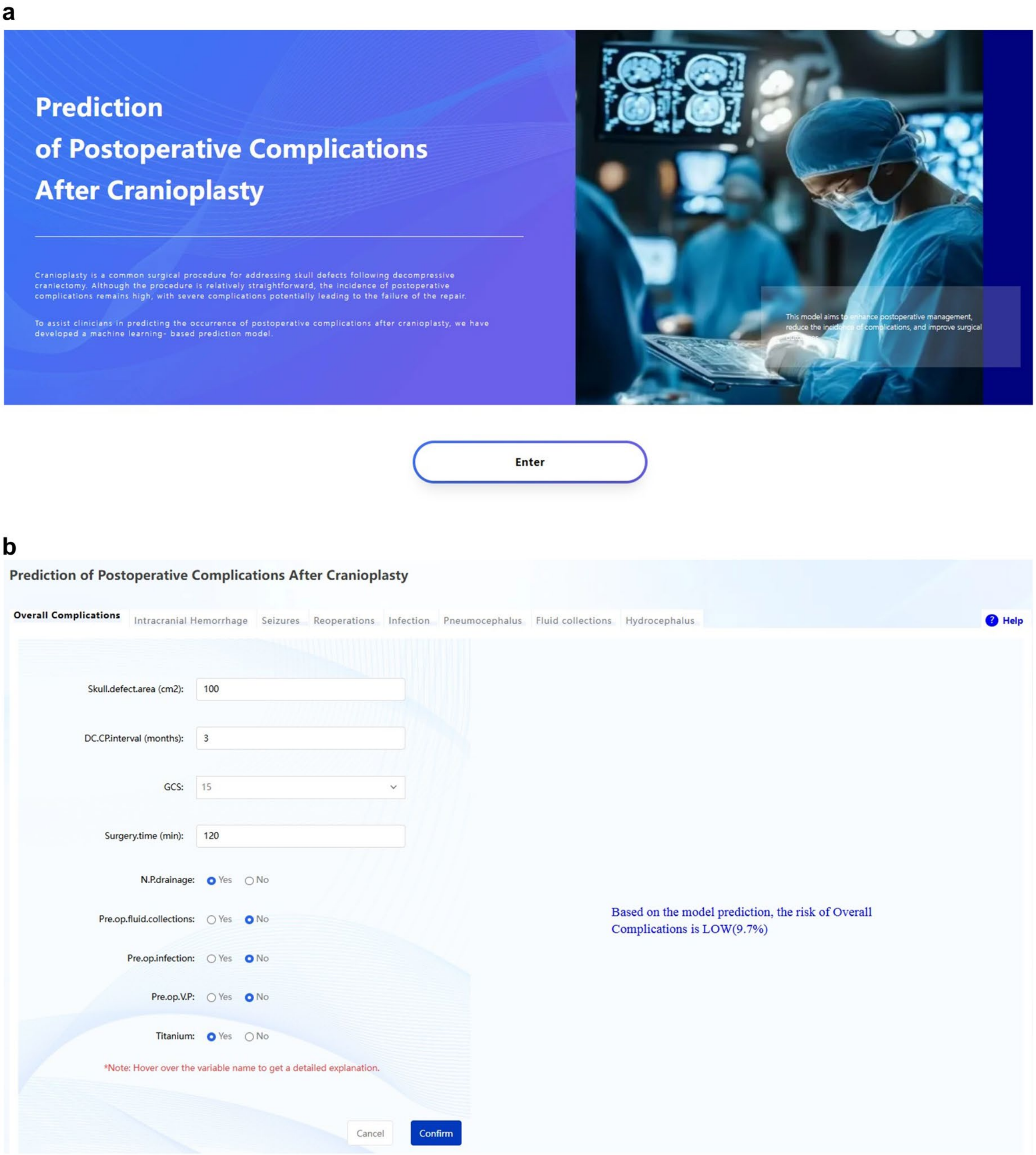

Figure S15. Web-based clinical decision-support platforms for postoperative complication risk prediction following cranioplasty.

(a) Landing page of the web application. **(b)** Main prediction interface with user inputs and risk output.
Note: To ensure reliable model performance in real-world clinical settings, the web-based tool includes built-in validation checks to assess input data quality. When predictor values are missing or fall outside plausible clinical ranges, the system automatically alerts the user and prevents prediction until the issue is resolved.

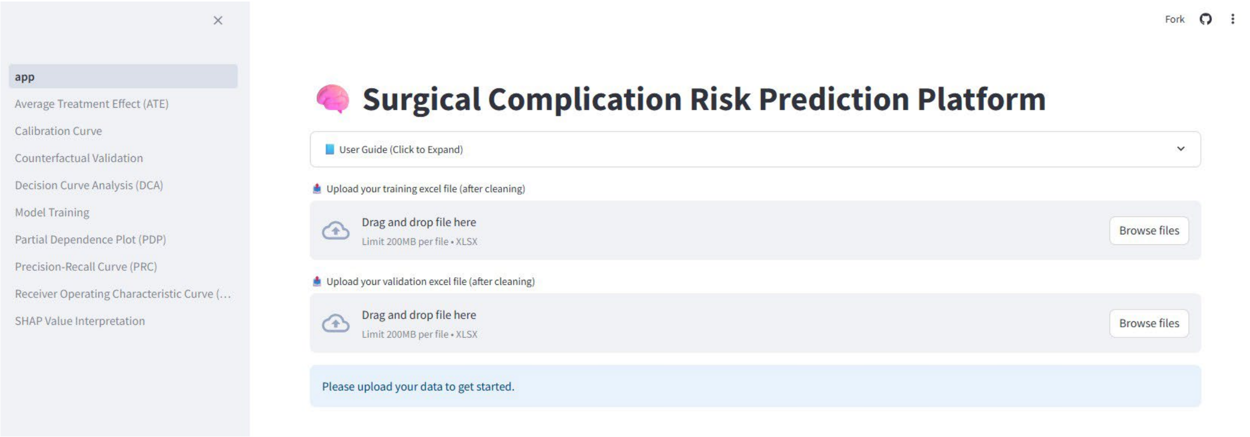

Figure S16. Generalizable methodological framework platform integrating model evaluation, interpretability, counterfactual, and causal inference analyses.
